# Ambient Air Pollution Exposure and Acute Osteoarthritis Exacerbations: A National Case-Crossover Analysis of 8 Million Outpatient Visits in China

**DOI:** 10.3390/toxics14010001

**Published:** 2025-12-19

**Authors:** Chao Li, Hong Zhang, Wenhui Chang, Yunlong Song, Yuchen Zhang, Ping Chen, Hongwei Zhang, Ge Li, Shaowei Wu

**Affiliations:** 1 Shaanxi Provincial Center for Disease Control and Prevention, Xi’an 710054, China; 2 Xijing Hospital, Air Force Military Medical University, Xi’an 710032, China; 3 Health Science Center, Xi’an Jiaotong University, Xi’an 710061, China; 4 Key Laboratory for Disease Prevention and Control and Health Promotion of Shaanxi Province, Xi’an 710061, China; 5 School of Humanities and Social Sciences, Xi’an Jiaotong University, Xi’an 710049, China; 6 Tuberculosis Hospital of Shaanxi Province, Xi’an 710100, China

**Keywords:** ambient air pollution, case crossover, outpatient visits, osteoarthritis, multi-pollutant mixtures

## Abstract

While the inflammatory properties of ambient air pollution may exacerbate osteoarthritis (OA), evidence on the population-level impact of multi-pollutant mixtures remains limited. This study quantifies the acute effects of short-term exposure to a complex mixture of six-criteria air pollutants on OA outpatient visits. In total, 8,146,141 OA visits from two national health insurance databases across 192 Chinese cities (2013–2017) were analyzed using a two-stage, time-stratified case-crossover design, combining conditional logistic regression with random-effects meta-analysis. The results showed that an interquartile range increase in the concentrations of PM_2.5_, PM_10_, NO_2_, SO_2_, O_3_, and CO was associated with significant increases in OA visits of 1.75%, 2.26%, 4.01%, 3.42%, 1.98%, and 1.87%, respectively. NO_2_ and SO_2_ demonstrated the strongest associations across OA subtypes. Multi-pollutant models confirmed that the risk of OA visits increased significantly under combined pollutant exposure. Population attributable fractions ranged from 2.15% for PM_2.5_ to 6.41% for NO_2_. This large-scale analysis provides novel evidence that transient exposure to complex pollution mixtures, rather than to individual pollutants, drives OA-related healthcare demand, with gaseous pollutants (NO_2_/SO_2_) being critical components. Our findings advocate for integrative air quality management strategies targeting co-emitted pollutants to mitigate OA exacerbations.

## 1. Introduction

Osteoarthritis (OA) constitutes a major disease burden globally, being a leading cause of chronic pain, functional limitations, and diminished quality of life [1,2]. Globally, OA affects over 500 million individuals, with its incidence escalating due to the rise in an aging demographic [3], presenting a significant risk to public health and contributing significantly to the global disease burden [4]. OA frequently coexists with other common chronic diseases in older adults [5], potentially exacerbating health risks. However, there are no effective treatments except for joint replacement. Therefore, it is beneficial to identify modifiable risk factors (such as environmental exposure) to mitigate OA-related burden.

Through experimental studies with animal models, it has been found that air pollution can induce oxidative stress, accelerating bone loss, increasing fracture risk and osteoporosis, and diminishing patient quality of life [6,7,8]. A recent study involving animals indicated that acute exposure to particulate matter, PM_2.5_ (aerodynamic diameter ≤ 2.5 μm) and PM_10_ (aerodynamic diameter ≤ 10 μm), independently or synergistically with gaseous air pollutants, such as NO_2_ (nitrogen dioxide), SO_2_ (sulfur dioxide), O_3_ (ozone), and CO (carbon monoxide), could elevate the levels of key biomarkers of OA, such as cartilage oligomeric matrix protein (COMP) and cytokines, which are show proof of a potential causal relationship between air pollutants and OA [9]. This suggests that increased air pollution may exacerbate the prevalence of OA. Therefore, to elucidate the relationship between air pollution and OA, more comprehensive and large-scale epidemiological studies with big data are needed.

Nevertheless, the connection between ambient air pollution and outpatient visits for OA remains poorly understood due to a scarcity of relevant evidence. Only a few time-series studies showed that short-term exposure to ambient air pollutants [10,11,12] is connected to a heightened outpatient service risk of OA. However, these studies are limited in scope, as their datasets only included one city or state, which could not accurately represent the overall picture. Additionally, these studies neglected its impact on OA subtypes. Moreover, previous research only focused on individual air pollutants and ignored the fact that particulate matter and gaseous pollutants are the primary constituents of the complex mixture known as ambient air pollution. As such, it is imperative to conduct a comprehensive assessment of the cumulative impact of multiple air pollutants [13,14]. Regrettably, no study thus far has evaluated the OA risk attributed to short-term joint exposure to multiple pollutants in the air.

The primary objective of this study was to assess the correlation between short-term exposure to key ambient air pollutants and outpatient visits for OA. This analysis, conducted using the Chinese national medical insurance database, encompassed OA overall and its five major subtypes: polyarthrosis, coxarthrosis, gonarthrosis, cervical degeneration, and lumbar degeneration. The excess risks (ERs) and the impact of ambient air pollution exposure on the burden of OA were assessed.

## 2. Materials and Methods

### 2.1. Outpatient Visits Database

City-specific data on OA-related outpatient visits were obtained from two major Chinese national health insurance databases for the period 2013–2017. Specifically, these are Urban Employee Basic Medical Insurance (UEBMI) and Urban Resident Basic Medical Insurance (URBMI), which cover 97.5% population of China by 2014 [15]. The state, together with society, enshrines the right of citizens to basic medical and healthcare services [16]. This study utilized anonymized, population-level health insurance data obtained through secure government partnerships for public health research purposes. All personally identifiable information was removed prior to analysis. Therefore, ethical review and individual informed consent were waived by the institutional review board, which is consistent with national guidelines and international standards for epidemiological studies using secondary de-identified data.

Initially, outpatient visit data for OA and its subtypes were extracted from 300 city-level administrative divisions, encompassing prefecture-level cities and above. Then, to ensure the consistency of the data and the robustness of the model, cities with total outpatient visits less than 50 were excluded during the study period. Finally, the study comprised a total of 192 prefecture-level cities or above. Figure S1 outlines the city selection process, while Figure S2 shows their central locations.

### 2.2. Ambient Air Pollution Dataset

Data on air pollutants were collected from the National Air Monitoring System (https://air.cnemc.cn:18007/ (accessed on 1 January 2021)). The ambient air pollution concentrations were averaged across all monitoring sites. Missing data in the included cities, accounting for a 13.29% missing rate, were supplemented using the Chinese Air Quality Reanalysis dataset (CAQRA) [17]. Meteorological data, such as relative humidity and daily average temperature, were collected from a Chinese meteorological database system (http://data.cma.cn/ (accessed on 1 January 2021)). Public holiday information was gathered from Chinese State Council General Office for 2013–2017. Data on the population and gross domestic product (GDP) per capita were derived from Chinese Urban Statistical Book of 2017.

### 2.3. Research Design

A two-stage, time-stratified case-crossover design [18,19] was employed to assess the relationships between short-term ambient air pollution exposure and outpatient visits for OA and its subtypes. In this method, the exposure of each case on the days preceding an acute event (case day) was compared with the exposure during the same year, month, and week (control day) when the event did not occur [20]. The potential confounding factors and long-term trend of unmeasured variables could be well controlled by this approach.

### 2.4. Statistical Analysis

Average air pollution concentrations at different time windows (single lag days and cumulative lag days), which are commonly used to capture the short-term acute effects of air pollution, were employed to examine the lag structure. Single lag days refer to the exposures of the current day and 1–2 days preceding admission. These are denoted as lag0, lag1, and lag2. Cumulative lag days are moving average exposures of the current day and 1–2 days prior to ambient air pollutants, which are denoted as lag01 and lag02. A conditional logistic regression model and random-effects model were utilized to estimate city-specific relationships between air pollutants and OA outpatient visits at different lag days [21].

To assess the variability of city-specific associations related to average air pollution concentrations per year, a scatter diagram was constructed. The total exposure–response (E-R) curves at representative lag time windows were generated. These curves were modeled using a natural cubic spline with knots at the first quartile, the median, and the third quartile. Subsequently, the results were aggregated using random-effect models [22]. To assess the excess risk and burden of outpatient visits linked to short-term exposure to air pollution, thresholds were set for excessive/heavily excessive concentrations among various air pollutants. These thresholds were based on the 24 h or 8 h average concentration limits of the AQG or ITs. The excess numbers of outpatient visits resulting from such exposure were then computed. This calculation helped us quantify the excess risks. To further evaluate the burden of OA outpatient visits due to air pollution, the attributable numbers (ANs) and attributable fractions (AFs) of OA outpatient visits were calculated. These metrics were based on the pooled associations with six ambient pollutants [23]. Additionally, the total excess numbers (ENs) [24] of OA outpatient visits were calculated.

To explore the combined effects of six major ambient air pollutants on OA daily outpatient visits, a supreme component analysis was initially performed. The first two principal components (eigenvalue > 1), which comprised the six ambient air pollutants, explained 73.75% of the total variance [25]. A weighted air pollution index was then derived by calculating the sum of the concentrations of the six air pollutants, each scaled by its respective weighting factor. Each pollutant concentration was weighted by single pollutant risk estimates (β coefficients) for daily outpatient visits due to OA [26]. The resulting air pollution score ranged from 3.28 to 418.85. The score signifies the relative magnitude of exposure to ambient air pollution. Based on this score, participants were further divided into four groups using the 25th, 50th, and 75th percentiles as cutoff points. The ENs were also calculated due to excessive exposure to ambient air pollution. Specifically, the higher quartiles of the air pollution score were compared to the lowest quartile. The formulas used for these calculations and the definitions are shown in Supplemental Materials.

Subgroup analyses were conducted based on several key factors. Gender was divided into male and female. Age groups were divided into <40, 40–64, 65–74, and ≥75 years. Seasons were defined as the warm season (April to September) and the cold season (October to March). For analytical purposes, the country was stratified into northern and southern regions, and insurance types were categorized as UEBMI and URBMI. In consideration of the substantial differences in meteorological features and air pollution characteristic between the northern and southern regions of China [27], cities were divided along the Qinling–Huaihe Line. To assess the difference between subgroups, the likelihood ratio test was used [28]. City-level information on the annual GDP per capita was obtained from city statistical yearbooks.

To ensure the robustness of the primary findings, a series of comprehensive sensitivity analyses was conducted. Firstly, two-pollutant models were applied to evaluate the stability of the observed relationships between ambient air pollutants and outpatient visits for OA, including both general cases and specific subtypes, while adjusting for the effects of other pollutants within the same main time window. Secondly, several key analytical parameters were adjusted: the degrees of freedom (df) for daily average temperature and relative humidity were varied from 3 to 6, and the time windows for calculating moving averages of these meteorological variables were tested across four durations: 7-day, 14-day, 21-day, and 28-day moving averages. Thirdly, the same model was reapplied across all 192 cities using both the UEBMI and URBMI datasets. Fourthly, associations were evaluated separately in two groups of cities: 108 cities with complete 5-year data and 84 cities with less than 5 years of data. Finally, the same model analyses were performed using ambient air pollution data obtained from a high-resolution air quality reanalysis dataset.

All statistical analyses were carried out in R software (Version 4.3.0) within the RStudio integrated development environment (Version 2022.12.0+353) [RStudio, PBC, Boston, MA, USA]. Primary findings were displayed as percent changes, with 95% confidence intervals (CIs) provided. These changes were linked to an interquartile range (IQR) uptick in ambient air pollutant concentrations. This approach was used to facilitate comparisons across different pollutants. Additionally, a threshold of two-sided *p* < 0.05 defined significance.

## 3. Results

### 3.1. Demographic Data and Air Pollution Profile

During the period from 2013 to 2017, in 192 Chinese cities, there were 8,146,141 outpatient visits for OA. Among the total OA visits, the specified subtypes—polyarthrosis, coxarthrosis, gonarthrosis, degeneration of cervical vertebra, and degeneration of lumbar spine—accounted for 4.15%, 1.24%, 31.84%, 11.70%, and 8.91%, respectively, while the remaining 42.2% were classified as unspecified osteoarthritis (Table 1).

In our research, the daily average concentrations were 49.50 μg/m^3^ for PM_2.5_, 81.00 μg/m^3^ for PM_10_, 27.90 μg/m^3^ for NO_2_, 23.20 μg/m^3^ for SO_2_, 78.80 μg/m^3^ for 8 h O_3_, and 0.90 mg/m^3^ for CO, respectively (Table 2). The degrees of correlation ranged from −0.33 to 0.87 among different air pollutants, temperature, and relative humidity (Figure S3). Table S1 exhibited the intraclass correlation coefficients for the daily concentrations of various air pollutants. These coefficients were calculated between data from the National Air Pollution Monitoring System and the CAQRA. Specifically, the coefficients ranged from 0.692 to 0.857.

### 3.2. Exposure Association and Risk Assessment

**Figure 1:**
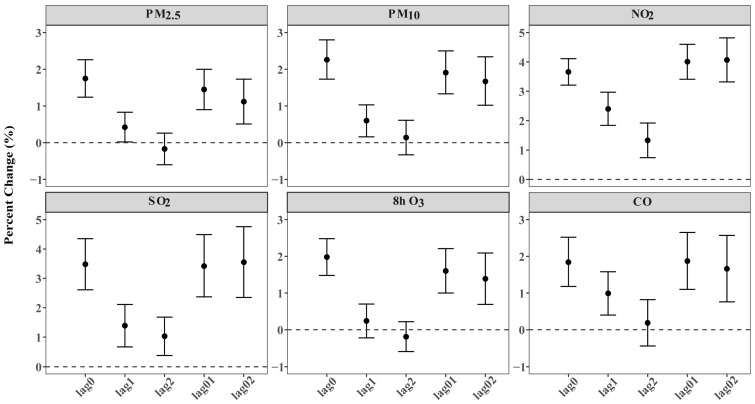
Overall percent changes with 95% confidence intervals in daily outpatient visits for osteoarthritis and its major subtypes associated with per-IQR increase in ambient air pollutants at different lag days in 192 Chinese cities, 2013–2017.

**Figure 2:**
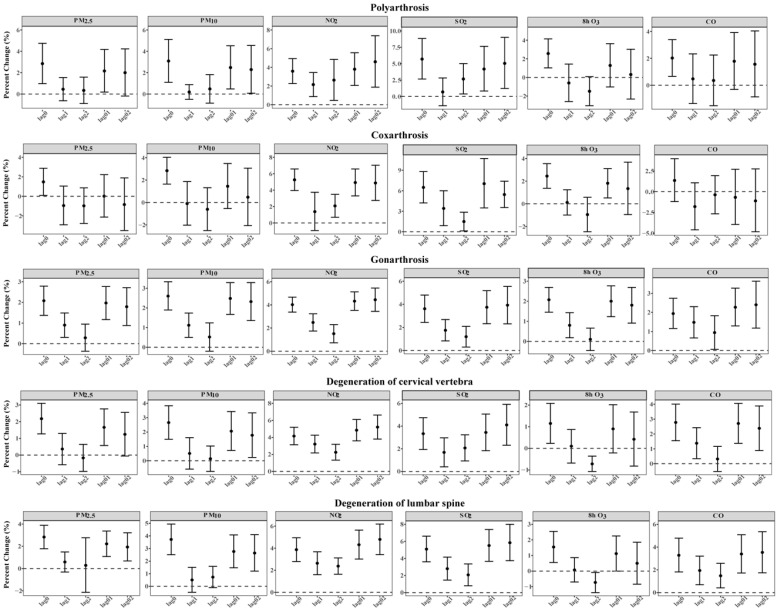
Overall percent changes with 95% confidence intervals in daily outpatient visits for major subtypes of osteoarthritis associated with per-IQR increase in ambient air pollutants at different lag days in 192 Chinese cities, 2013–2017. Outpatient visits data from 120, 143, 182, 112, and 157 cities were eligible to generate valid effect estimates for polyarthrosis, coxarthrosis, gonarthrosis, degeneration of cervical vertebra, and degeneration of lumbar spine, respectively.

**Figure 3:**
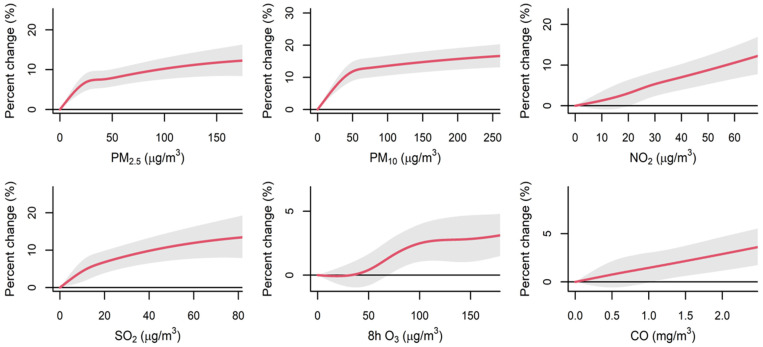
Overall exposure–response curves for short-term exposures to ambient air pollutants at the main time window ^a^ and percent changes (%) in daily outpatient visits for osteoarthritis in 192 Chinese cities, 2013–2017 ^b^. ^a^ The main time window was lag0 for PM_2.5_, PM_10_, and 8 h O_3_ and lag01 for NO_2_, SO_2_, and CO. ^b^ The solid red lines indicate the estimated percent changes in daily outpatient visits for osteoarthritis, and the shaded regions indicate 95% confidence intervals.

Figure 1 depicts the overall percent changes in OA outpatient visits corresponding to the per IQR increment in air pollutant levels at distinct lag days, and the strongest effects of OA outpatient visits for PM_2.5_, PM_10_, and 8 h O_3_ appeared on the current day (lag 0). Outpatient risks for OA increased 1.75% (95% CI: 1.24%, 2.26%), 2.26% (95% CI: 1.73%, 2.80%), and 1.98% (95% CI: 1.48%, 2.48%) by per-IQR increase, correspondingly, while for NO_2_, SO_2_, and CO, the strongest effects were at lag 01. Outpatient risks for OA increased 4.01% (95% CI: 3.41%, 4.605%), 3.42% (95% CI: 2.37%, 4.49%), and 1.87% (95% CI: 1.10%, 2.65%) by per-IQR increase, correspondingly. Additionally, along the extension of a single lag day, percent change gradually decreases, and with the extension of cumulative lag days, percent change shows an increasing trend. The estimated effects of ambient air pollutants’ per-unit increase in exposure concentrations were consistent with the effects per IQR increase, and both suggested a more apparent association for NO_2_ and SO_2_ when compared with the other air pollutants (Table S2). Associations at the city level are shown in Figure S4. Figure 2 indicates that short-term exposures to NO_2_ and SO_2_ had a significant impact. This impact was reflected in increased daily outpatient visits for all major OA subtypes, which were consistent with the results for total OA. Moreover, consistent with the results for 8 h O_3_, the strongest associations between 24 h O_3_ and daily outpatient visits for OA appeared at the current day of OA outpatient visits (lag 0) (Figure S9). The E-R curves for different ambient air pollutants generally appeared to be linear, and no safe threshold was observed for any ambient air pollutant, as presented in Figure 3.

### 3.3. Excessive Risk and Modified Effect

Table 3 shows that the overall ERs in outpatient visits for OA ranged from 1.59% (95% CI: 1.21–1.97%), in association with heavily excessive CO (≥1.5 mg/m^3^ vs. <1.15 mg/m^3^), to 7.42% (95% CI: 7.01–7.82%), in association with heavily excessive PM_10_ (≥150 μg/m^3^ vs. <50 μg/m^3^), compared to ambient air pollution concentrations below the AQG or ITs (below 1.15 mg/m^3^ for CO concentrations). In addition, heavily excessive levels of ambient air pollution were typically linked to significantly higher risk estimates compared to excessive air pollution concentrations for different air pollutants across various lag days, except for 8 h O_3_ (Figure S5).

**Figure 4:**
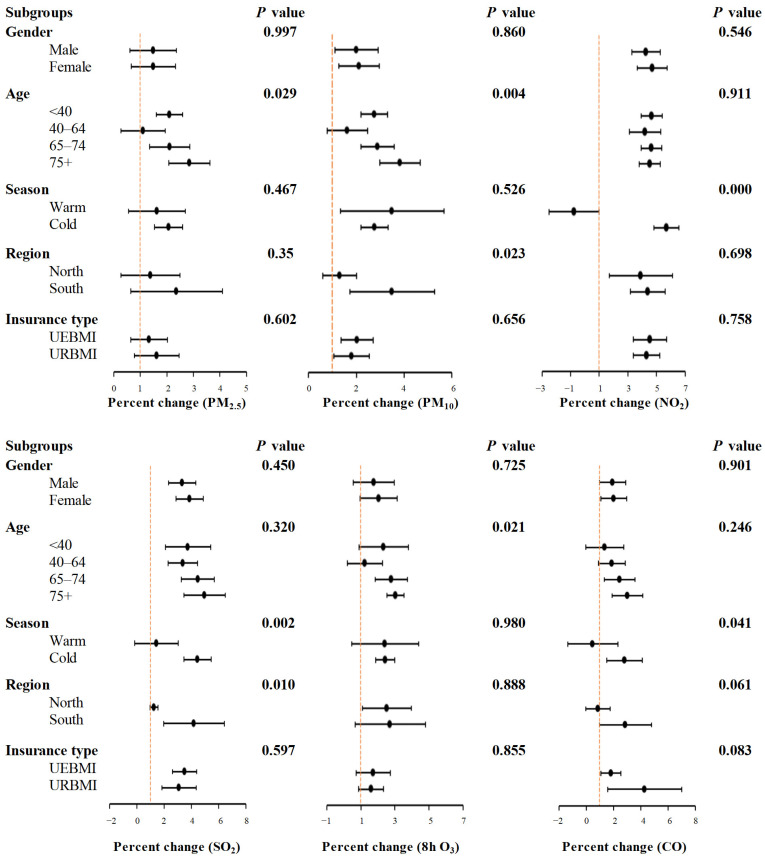
Percent changes with 95% confidence intervals in daily outpatient visits for osteoarthritis per IQR increase in ambient air pollutants at the main time window ^a^ stratified by gender, age, season, region, and insurance type in 192 Chinese cities, 2013–2017. ^a^ The main time window was lag0 for PM_2.5_, PM_10_, and O_3_ and lag01 for NO_2_, SO_2_, and CO; the *p* value indicates the statistical difference between subgroups.

Figure 4 presents the pooled associations. The analysis was conducted across various subgroups and focused on the main time windows. The associations varied across the four age groups. For PM_2.5_, PM_10_, and 8 h O_3_, significant differences were observed (*P_difference_* < 0.05). Moreover, these associations were generally more apparent in the elders with age ≥ 75 years. In addition, for NO_2_, SO_2_, and CO, the associations were more pronounced in the cold season compared to the warm season. Additionally, geographical variations were noted for PM_10_ and SO_2_, since the associations were stronger in south China than in north China, respectively (all *P_difference_* < 0.05). No significant association differences were found between subgroups divided by gender and insurance type. Meta-regression models were used to explore the potential modifying effects of individual and city-level variables. These models revealed several key findings. For NO_2_, SO_2_, and CO, the associations were found to be stronger in the cold season. In contrast, these associations were weaker in the warm season (all *P_difference_* < 0.05) (Table S3).

**Figure 5:**
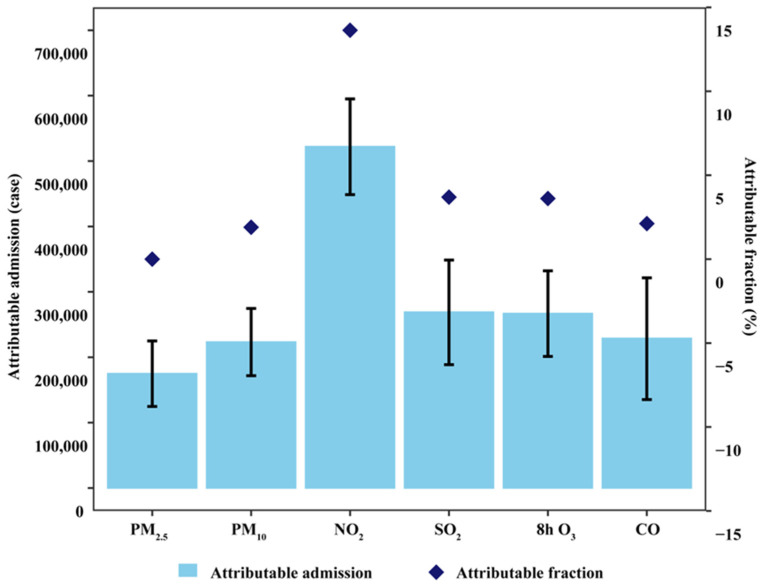
Attributable numbers and fractions of outpatient visits with 95% confidence intervals for osteoarthritis associated with short-term exposures to ambient air pollutants at the main time window ^a^ in 192 Chinese cities, 2013–2017. ^a^ The main time window was lag0 for PM_2.5_, PM_10_, and 8 h O_3_ and lag01 for NO_2_, SO_2_, and CO.

The ANs of OA-related outpatient visits ranged from 175,021 (95% CI: 124,782–224,918) for PM_2.5_ to 522,156 (95% CI: 448,597–594,963) for NO_2_, and the AFs ranged from 2.15% (95% CI: 1.53–2.76%) for PM_2.5_ to 6.41% (95% CI: 5.51–7.30%) for NO_2_ during the study period (Figure 5). During the study period, the highest excess fractions (EFs) of OA-related outpatient visits were identified for PM_10_. For excessive ambient air pollution to PM_10_, the EF amount was 2.65% (95% CI: 2.50–2.80%). For heavily excessive exposure to PM_10_, the EF amount was 0.87% (95% CI: 0.83–0.92%). These results are illustrated in Figure S6.

### 3.4. Combined Effect and Sensitivity Analyses

**Figure 6:**
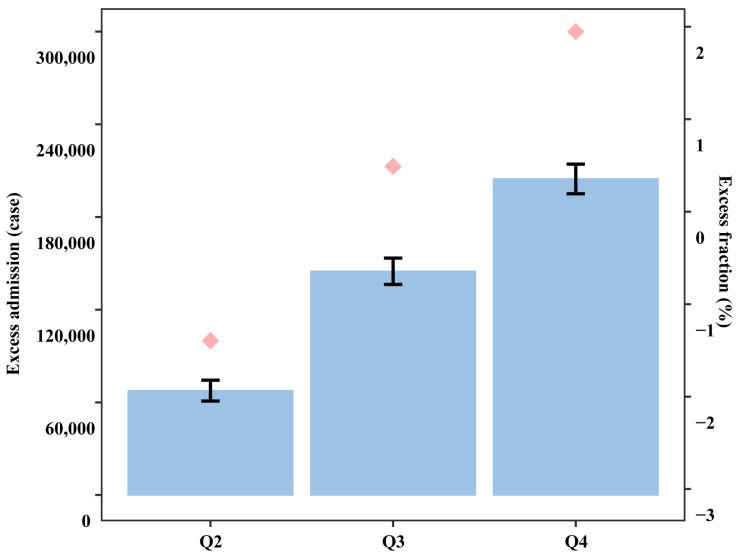
Excess numbers and fractions with 95% confidence intervals in daily outpatient visits for osteoarthritis associated with excessive ambient air pollution score ^a^ in 192 Chinese cities, 2013–2017. ^a^ According to the quartiles of the air pollution score, Q_2_ ranged from 18.30 to 25.60, Q_3_ ranged from 25.61 to 35.80, and Q_4_ was larger than or equal to 35.81; Q_1_ (<18.30) was defined as the reference category.

Table 4 shows the associations between air pollution scores and outpatient visits for OA. When comparing the highest air pollution score quartile (Q4 ≥ 35.81) to the lowest quartile (Q1 < 18.30), an increasing trend in the risk of OA-related outpatient visits was observed across higher air pollution score quartiles. The *p* value for this trend was less than 0.001. Specifically, the EF of OA-related outpatient visits in the highest quartile was 2.51% (95% CI: 2.39–2.63%) compared to the lowest quartile, respectively (Figure 6).

Sensitivity analyses were conducted to assess the robustness of the associations between air pollutants and OA-related outpatient visits. The results showed that the associations remained unchanged in two-pollutant models for PM_10_, NO_2,_ and 8 h O_3_ with OA-related outpatient visits. However, when NO_2_ was controlled for in two-pollutant models, the association for CO became inverse, and the associations for PM_2.5_ and SO_2_ lost statistical significance (Table S4). The study observed the associations between ambient air pollutants and outpatient visits for OA subtypes. Some changes were noted in these associations. However, the strongest associations generally remained unchanged in two-pollutant models. These results are presented in Table S5. Subsequently, the model was re-run with several modifications. The degrees of freedom for average temperature and relative humidity were altered, and the time windows for calculating the moving averages of these variables were adjusted. Furthermore, the model was fitted using data from 108 Chinese cities with complete 5-year health records, while ambient air pollution data were sourced from the Chinese Air Quality Reanalysis dataset (CAQRA). Despite these adjustments, the results remained largely stable. These findings are presented in Table S6 and Figures S7 and S8.

## 4. Discussion

This case-crossover study, to our knowledge, used the largest dataset to date for investigating the association between ambient air pollution and OA outpatient visits. It comprehensively assessed the associations between short-term air pollution exposure and outpatient visits for OA and its major subtypes. It is the first time to provide nationwide evidence on the outpatient visits burden of OA associated with ambient air pollution. Overall, using national insurance outpatient records, short-term exposure to six key ambient air pollutants was analyzed. Significant associations with increased outpatient visits were identified for OA and its major subtypes. In addition, the associations for NO_2_ exhibited the most robust among the major air pollutants across various regression models. The particularly strong association observed for NO_2_ may be explained by its role in systemic inflammation and oxidative stress pathways relevant to OA pathophysiology. Experimental studies suggest that nitrogen dioxide can induce pulmonary and systemic inflammation, leading to increased circulation of pro-inflammatory cytokines such as interleukin-6 (IL-6) and tumor necrosis factor-alpha (TNF-α) [29]. These cytokines are known to promote synovitis, cartilage degradation, and pain sensitization in osteoarthritis joints [2]. Furthermore, NO_2_ exposure can enhance oxidative stress, potentially accelerating cellular damage in joint tissues [6,7]. This proposed mechanism, linking inhaled pollutant to systemic inflammation and joint tissue response, provides a plausible biological basis for our epidemiological findings.

There was no safe threshold observed for any ambient air pollutant in the exposure–response curve. When compared with exposure to relatively low concentrations of air pollutants, exposure to excessive/heavily excessive concentrations (defined using the WHO AQG or its ITs) of air pollutants was linked to considerable ERs in OA-related outpatient visits. Additionally, OA-related outpatient visit risk increased significantly under the combined effects of multi-pollutant exposure. The ambient air pollution in most districts seriously exceeds the WHO AQG standard [30], which suggest that stricter measures and policies should be tried to avoid exposure to high-level ambient air pollution, thereby lowering OA-related risk.

Earlier studies have chiefly focused on the relationship between ambient air pollutants and rheumatic arthritis (RA) [29,31,32], with scant studies exploring the connection with OA. A recent systematic review identified novel OA-related modifiable risk factors, including heavy metals and persistent organic pollutants [33]. However, the review failed to consider the health consequences of ambient air pollutants. A study in a selected Taiwan, China hospital used a multivariate disease–air pollution model to evaluate the risks of outpatient visits for different diseases. The study focused on the impact of concurrent short-term exposure to multiple ambient air pollutants and found that patients exposed to higher levels of air pollution had a higher risk of OA-related outpatient visits [34]. However, the study did not provide quantitative risk estimates for OA-related outpatient visits. Specifically, it did not cover estimates tied to per-unit rises in each air pollutant. A time-series analysis was conducted in Central–Eastern China. It covered 26 cities in Anhui province. For PM_2.5_, a per 10 μg/m^3^ increase was associated with a relative risk (RR) of 1.023 for OA outpatient visits. The 95% CI was 1.005–1.041. For PM_10_, the RR was 1.011, with a 95% CI of 1.001–1.025. These elevated risks were observed at the lag 2 day [11]. Another time-series study was conducted using data collected in Beijing. It investigated the cumulative relationships and discovered that a per-unit increase in various air pollutants at specific time windows corresponded to varying levels of rises in daily OA-related outpatient visits. Additionally, a substantial amount of OA-related outpatient visits was attributed to short-term exposure to air pollution [12]. However, the data of the two studies was limited to specific cities, lacking representativeness. A British birth cohort investigation [35], a cross-sectional survey conducted across the United States [36], and a time-series study in France [37] all supported the view that long-term exposure to ambient air pollution was associated with OA.

Different from previous studies [38], national outpatient visit data were utilized to reach the goal of our study for the first time. This approach allowed us to better capture the chronological sequence between ambient air pollution exposure and the disease clinical manifestations. Based on the national outpatient visit data of the Chinese urban population, our study provides more convincing evidence that short-term exposure to major ambient air pollutants is linked to an increased risk of OA. Consistent results were found by employing an air pollution score to explore the combined effects of these pollutants, echoing earlier research on the health effects of short-term exposure to ambient particulate matter and gaseous pollutants. These findings echo earlier research on the health effects of short-term exposure to ambient PM and gaseous air pollutants [39]. Specifically, the E-R curves in our study showed a consistent increasing trend between different air pollutants and OA-related outpatient visits, with no evidence of a safe threshold. A significantly higher risk of OA outpatient visits in the higher quartiles of the air pollution score compared to the lowest quartile was observed. Furthermore, a strong association between short-term exposure to the six major pollutants and OA-related outpatient visit risk was demonstrated. The overall percent change in OA-related outpatient visits associated with short-term exposure to heavily excessive concentrations of air pollutants was much stronger than that associated with lower concentrations.

To our knowledge, this is the first study to provide comprehensive evaluation of the potential acute effects of short-term exposure to ambient air pollution on increased outpatient visits for OA subtypes. Among the five subtypes, gonarthrosis has the largest number of outpatient visits, and it is also most consistently associated with different ambient air pollutants. Two previous studies about the incidence of OA subtypes in central Massachusetts [40] and in a Southern European/Mediterranean nation (Catalonia) [41] both reported that the incidence rate for gonarthrosis was higher than the other subtypes. Accordingly, a single time-series study on Beijing revealed that a per-unit increase in PM_2.5_ was related to a 1.41% (95% CI: 1.40–1.41%) increase in gonarthrosis outpatient visits [10]. However, that study did not assess the associations between other major air pollutants and OA outpatient visits. Our study expands on this by examining the impact of six key air pollutants. The analysis found that short-term exposure to each of these pollutants was associated with increased outpatient visits for gonarthrosis. This finding provides a more comprehensive and novel understanding of how ambient air pollution affects this condition. Collectively, these findings, derived from more comprehensive health data, offer stronger evidence for the acute effects of short-term ambient air pollution exposure on outpatient visit risk for OA subtypes.

This research has both strengths and limitations. Regarding its strengths, to our knowledge, it is the largest and most comprehensive study to date examining the associations between six major ambient air pollutants and daily outpatient visits for OA and its major subtypes. In contrast to our previous study, which assessed hospital admissions for OA [42], the present study utilized national outpatient visit data to reach the goal of our study for the first time. This study utilized a nationwide representative dataset. A case-crossover design was also employed, effectively controlling for time-invariant confounders at both the individual level (e.g., age, gender, and socioeconomic status) and city level (e.g., population density). Furthermore, this study assessed the combined effects associated with outpatient visit risk and burden attributable to multi-pollutant exposure, particularly at concentrations exceeding relevant Air Quality Guidelines (AQGs). Regarding its limitations, firstly, because individual-level information is infeasible given the large scale of the study, the use of city-level average exposure may lead to potential misclassification of exposure. Secondly, there is a possibility of diagnostic errors in OA and its subtypes. This could introduce misclassification bias. While it is unlikely to change the overall associations between air pollution and outpatient visits, it may reduce the accuracy of the effect size estimates. Thirdly, the current dataset only included urban residents; thus, the generalization of the results needed to be cautious.

The findings of this national study carry significant implications for public health policy and air quality management. First, the consistent associations, especially with gaseous pollutants (NO_2_ and SO_2_), underscore the need for integrated air quality control strategies that target co-emitted pollutants from common sources like fossil fuel combustion rather than focusing on single pollutants. Second, the absence of a safe threshold and the elevated risks at concentrations exceeding WHO AQGs highlight the potential public health benefits of adopting stricter air quality standards aligned with the latest WHO guidelines. Third, the significant population attributable fractions suggest that reducing ambient air pollution could substantially reduce the healthcare burden from OA exacerbations. Policymakers could consider targeted health advisories for susceptible populations (e.g., the elderly) during periods of high pollution alongside long-term strategies for emission reduction. These measures would not only benefit OA patients but also likely reduce the incidence of other air pollution-related conditions.

## 5. Conclusions

This nationwide case-crossover analysis provides robust evidence that short-term exposure to six major ambient air pollutants is significantly associated with increased risks of outpatient visits for overall OA and its major subtypes, particularly gonarthrosis. Among the pollutants, NO_2_ and SO_2_ showed the strongest and most consistent associations. Exposure–response relationships appeared linear without evidence of a safe threshold. A significant proportion of OA outpatient visits were attributable to short-term air pollution exposure. However, this study has limitations, including the use of city-level rather than individual-level exposure estimates, potential diagnostic misclassification of OA subtypes, and a focus on the urban insured population, which may limit generalizability to rural areas. Future studies would benefit from employing individual-level exposure assessment techniques (e.g., personal monitors or high-resolution models), incorporating clinical biomarker data to explore underlying mechanisms, and extending research to rural and uninsured populations. Investigating the joint effects of pollution with other environmental or lifestyle factors could also provide a more holistic understanding of OA exacerbations. Notwithstanding these limitations, our findings strengthen the evidence base for the acute effects of air pollution on OA and support the need for comprehensive air quality management strategies to mitigate this modifiable environmental risk factor.

## Figures and Tables

**Table 1:** Descriptive statistics of daily outpatient visits for osteoarthritis and its major subtypes in 192 Chinese cities of prefecture level or above, 2013–2017.

Osteoarthritis	No. (%) of Daily Outpatient Visits
Total	8,146,141
Insurance type	
UEBMI	7,508,631 (92.17)
URBMI	637,510 (7.83)
Subtype	
Polyarthrosis	338,259 (4.15)
Coxarthrosis	101,022 (1.24)
Gonarthrosis	2,593,480 (31.84)
Degeneration of cervical vertebra	952,888 (11.70)
Degeneration of lumbar spine	725,983 (8.91)
Unspecified osteoarthritis ^a^	3,434,509 (42.2)

^a^ Unspecified osteoarthritis was not included in the analysis. Abbreviations: UEBMI, urban employee-based basic medical insurance scheme; URBMI, urban resident-based basic medical insurance scheme.

**Table 2:** Descriptive statistics of daily ambient air pollutants ^a^ and meteorological factors in 192 Chinese cities, 2013–2017.

Variable	Mean			SD	IQR	Q_1_	Q_99_
	Total	Season: Warm/Cold ^b^	Region: North/South ^c^				
Ambient air pollutant ^a^							
PM_2.5_ (μg/m^3^)	49.50	37.10/61.90	52.80/46.80	39.60	38.30	4.90	200.60
PM_10_ (μg/m^3^)	81.00	65.70/96.40	93.20/70.90	61.00	61.00	6.60	294.80
NO_2_ (μg/m^3^)	27.90	22.50/33.30	30.20/25.90	18.30	22.90	1.00	84.40
SO_2_ (μg/m^3^)	23.20	16.40/30.00	29.00/18.40	23.70	18.00	1.10	117.50
8 h O_3_ (μg/m^3^)	78.80	95.20/62.30	80.40/77.50	37.20	49.10	12.40	184.20
CO (mg/m^3^)	0.90	0.80/1.10	1.10/0.90	0.60	0.60	0.10	3.10
Meteorological factor ^a^							
Temperature (°C)	14.10	21.50/6.60	9.80/17.60	11.10	15.80	−16.60	31.60
Relative humidity (%)	68.40	70.20/66.60	59.10/76.10	18.50	25.00	21.00	99.00

^a^ The 24 h average concentrations were used for PM_2.5_, PM_10_, NO_2_, SO_2_, and CO, daily 8 h maximum for O_3_, and 24 h average values for temperature and relative humidity. ^b^ Warm season: April to September; cold season: October to March. ^c^ The 192 cities included in the study were classified into the north (*n* = 89) and south (*n* = 103) regions divided by the Qinling Mountains–Huai River Line.

**Table 3:** Overall percent changes with 95% confidence intervals in daily outpatient visits for osteoarthritis associated with excessive or heavily excessive air pollution concentrations under different definitions at the main time window ^a^ in 192 Chinese cities, 2013–2017.

Air Pollutant	Reference Concentration	Excessive/Heavily Excessive Concentration	Percent Change (95% CI) ^b^
PM_2.5_	<25 μg/m^3 c^	≥25 μg/m^3^	2.78 (2.56, 3.00)
		25–74 μg/m^3^	2.28 (2.06, 2.51)
		≥75 μg/m^3 e^	4.93 (4.60, 5.26)
PM_10_	<50 μg/m^3 d^	≥50 μg/m^3^	4.00 (3.79, 4.21)
		50–149 μg/m^3^	3.50 (3.28, 3.71)
		≥150 μg/m^3 e^	7.42 (7.01, 7.82)
NO_2_	<25 μg/m^3 f^	≥25 μg/m^3^	2.67 (2.37, 2.97)
		25–49 μg/m^3^	2.29 (2.00, 2.59)
		≥50 μg/m^3 g^	6.28 (5.90, 6.67)
SO_2_	<20 μg/m^3 h^	≥20 μg/m^3^	1.90 (1.65, 2.16)
		20–39 μg/m^3^	1.75 (1.50, 2.00)
		≥40 μg/m^3 f^	3.80 (3.26, 4.34)
8 h O_3_	<70 μg/m^3 i^	≥70 μg/m^3^	3.02 (2.81, 3.23)
		70–99 μg/m^3^	1.95 (1.72, 2.19)
		≥100 μg/m^3 f^	3.52 (3.26, 3.79)
CO	<1.15 mg/m^3 j^	≥1.15 mg/m^3^	1.17 (0.91, 1.43)
		1.15–1.49 mg/m^3^	0.90 (0.62, 1.19)
		≥1.5 mg/m^3 k^	1.59 (1.21, 1.97)

^a^ The main time window was lag0 for PM_2.5_, PM_10_, and 8 h O_3_ and lag01 for NO_2_, SO_2_, and CO. ^b^ The reference category for each air pollutant was the days with low daily air pollutant concentrations. ^c^ The Chinese Ambient Air Quality Standards class I (24 h average), which was based on the interim target-4 level of WHO AQG-2005 (24 h average). ^d^ The interim target-4 level of WHO AQG-2021 (24 h average). ^e^ The interim target-1 level of WHO AQG-2021 (24 h average). ^f^ The WHO AQG-2021 level (24 h average for NO_2_, and SO_2_; 8 h maximum for O_3_). ^g^ The interim target-2 level of WHO AQG-2021 (24 h average). ^h^ The WHO AQG-2005 level (24 h average). ^i^ Non-anthropogenic level. ^j^ A systematic review study (referenced by the AQG-2021 report) showed that the median level of short-term exposure to CO reported by most studies was below 1.15 mg/m^3^. ^k^ Similar health effects of CO were observed when the cutoff value of CO concentration was set to 1.50 mg/m^3^ compared to 4 mg/m^3^ (the WHO AQG-2021 level) according to the previous literature.

**Table 4:** Percent changes (%) with 95% confidence intervals of daily outpatient visits for osteoarthritis associated with the air pollution score in 192 Chinese cities, 2013–2017.

Air Pollution Score (Quartiles)	Percent Change (95% CI)	*p* Value	*p* for Trend
Q_1_ (<18.30)	Reference		
Q_2_ (18.30–25.60)	2.81 (2.53, 3.10)	<0.001	<0.001
Q_3_ (25.61–35.80)	5.18 (4.88, 5.49)	<0.001	
Q_4_ (≥35.81)	7.30 (6.96, 7.64)	<0.001	

## Data Availability

The original contributions presented in this study are included in the article/Supplementary Material. Further inquiries can be directed to the corresponding authors.

## Supplementary Materials

The following supporting information can be downloaded at: https://www.mdpi.com/article/10.3390/toxics14010001/s1, Table S1. Comparison between ambient air pollution data from the high-resolution air quality reanalysis dataset over China and ambient air pollution data from the National Urban Air Quality Real-time Publishing Platform. Table S2. Overall percent changes and 95% confidence intervals in daily outpatient visits for osteoarthritis associated with per unit increase in ambient air pollutants at the main time window a in 192 Chinese cities, 2013–2017. Table S3. Meta-regression results of individual and city-specific variables for the significant associations between daily outpatient visits for osteoarthritis and per IQR increase in ambient air pollutants at the main time window a. Table S4. Overall percent changes with 95% confidence intervals in daily outpatient visits for osteoarthritis associated with per IQR increase in ambient air pollutants in two-pollutant models at the main time window a in 192 Chinese cities, 2013–2017. Table S5. Overall percent changes with 95% confidence intervals in daily outpatient visits for subtypes of osteoarthritis associated with per IQR increase in ambient air pollutants in two-pollutant models at the main time window a in 192 Chinese cities, 2013–2017. Table S6. Sensitivity analyses for the associations between per IQR increase in ambient air pollutant concentrations at the main time window a and daily outpatient visits for osteoarthritis in 192 Chinese cities, 2013–2017. Figure S1. Cities selection flow chart. Figure S2. Central locations of the 192 Chinese cities of prefecture-level or above included in the study. Figure S3. Spearman’s correlation coefficients between ambient air pollutants and meteorological factors in 192 Chinese cities, 2013–2017. Figure S4. Scatter plots of city-specific percent changes in daily outpatient visits for osteoarthritis associated with per IQR increase in PM2.5 (A), PM10 (B), NO2 (C), SO2 (D), 8h O3 (E), and CO (F) at the main time window (Y axis) versus the annual average air pollutant concentrations at city level (X axis). Figure S5. Overall percent changes with 95% confidence intervals in daily outpatient visits for osteoarthritis associated with excessive or heavily excessive ambient air pollutant concentrations under different definitions at different lag days in 192 Chinese cities, 2013–2017. Figure S6. Excess numbers and fractions with 95% confidence intervals in outpatient visits for osteoarthritis associated with excessive or heavily excessive ambient air pollutant concentrations under different definitions a at the main time window b in 192 Chinese cities, 2013–2017 c. Figure S7. Overall percent changes with 95% confidence intervals in daily outpatient visits for osteoarthritis per IQR increase in ambient air pollutants at different lag days in 108 Chinese cities with 5-year data (A) and 84 Chinese cities with data less than 5 years (B), 2013–2017. Figure S8. Overall percent changes with 95% confidence intervals in daily outpatient visits for osteoarthritis per IQR increase in ambient air pollutants at different lag days in 192 Chinese cities, 2013–2017, using ambient air pollution data from the high-resolution air quality reanalysis dataset over China. Figure S9. Overall percent changes and 95% confidence intervals in daily outpatient visits for osteoarthritis with per IQR increase in 24h average O3 at different lag days in 192 Chinese cities, 2013–2017.

## Abbreviations

The following abbreviations are used in this manuscript:

8 h O_3_	8 h maximum ozone
CO	carbon monoxide
IQR	interquartile range
NO_2_	nitrogen dioxide
PM_2.5_	particulate matter ≤ 2.5 μm in aerodynamic diameter
PM_10_	particulate matter ≤ 10 μm in aerodynamic diameter
Q_1_	1st percentile
Q_2_	2nd quartile
Q_3_	3rd quartile
Q_4_	4th quartile
Q_99_	99th percentile
SD	standard deviation
SO_2_	sulfur dioxide
UEBMI	urban employee-based basic medical insurance scheme
URBMI	urban resident-based basic medical insurance scheme
