# Supplementary material for: Ambient Air Pollution Exposure and Acute Osteoarthritis Exacerbations: A National Case-Crossover Analysis of 8 Million Outpatient Visits in China
Source: Toxics. 2025 Dec 19;14(1):1. doi: 10.3390/toxics14010001 (PMC12845690; doi:10.3390/toxics14010001)
Supplement: Supplementary file 1 [file toxics-14-00001-s001.zip › toxics-4007113-supplementary.pdf]

Supplementary Material

Ambient air pollution exposure and acute osteoarthritis exacerbations: A national case-crossover analysis of 8 million outpatient visits in China

Chao Li, Hong Zhang, Wenhui Chang, Yunlong Song, Yuchen Zhang, Ping Chen, Hong Zhang, Ge Li, Shaowei Wu

Supplementary definition of the formula

Table of contents

Tables

Table S1. Comparison between ambient air pollution data from the high-resolution air quality reanalysis dataset for China and ambient air pollution data from the National Urban Air Quality Real-time Publishing Platform.

Table S2. Overall percent changes and 95% confidence intervals in daily outpatient visits for osteoarthritis associated with per-unit increase in ambient air pollutants at the main time window [a] in 192 Chinese cities, 2013-2017.

Table S3. Meta-regression results of individual and city-specific variables for the significant associations between daily outpatient visits for osteoarthritis and per IQR increase in ambient air pollutants at the main time window [a].

Table S4. Overall percent changes with 95% confidence intervals in daily outpatient visits for osteoarthritis associated with per-IQR increase in ambient air pollutants in two-pollutant models at the main time window [a] in 192 Chinese cities, 2013-2017.

Table S5. Overall percent changes with 95% confidence intervals in daily outpatient visits for subtypes of osteoarthritis associated with per-IQR increase in ambient air pollutants in two-pollutant models at the main time window [a] in 192 Chinese cities, 2013-2017.

Table S6. Sensitivity analyses for the associations between per-IQR increase in ambient air pollutant concentrations at the main time window [a] and daily outpatient visits for osteoarthritis in 192 Chinese cities, 2013–2017.

Figures

Figure S1. City selection flow chart.

Figure S2. Central locations of the 192 Chinese cities at the prefecture level or above included in the study.

Figure S3. Spearman's correlation coefficients between ambient air pollutants and meteorological factors in 192 Chinese cities, 2013-2017.

Figure S4. Scatter plots of city-specific percent changes in daily outpatient visits for osteoarthritis associated with per-IQR increase in $PM_{2.5}$ (A), PM_{10} (B), NO_2 (C), SO_2 (D), 8h O_3 (E), and CO (F)

at the main time window (Y axis) versus the annual average air pollutant concentrations at the city level (X axis).

Figure S5. Overall percent changes with 95% confidence intervals in daily outpatient visits for osteoarthritis associated with excessive or heavily excessive ambient air pollutant concentrations under different definitions at different lag days in 192 Chinese cities, 2013-2017.

Figure S6. Excess numbers and fractions with 95% confidence intervals in outpatient visits for osteoarthritis associated with excessive or heavily excessive ambient air pollutant concentrations under different definitions [a] at the main time window [b] in 192 Chinese cities, 2013-2017 [c].

Figure S7. Overall percent changes with 95% confidence intervals in daily outpatient visits for osteoarthritis per IQR increase in ambient air pollutants at different lag days in 108 Chinese cities with 5-year data (A) and 84 Chinese cities with data for less than 5 years (B), 2013-2017.

Figure S8. Overall percent changes with 95% confidence intervals in daily outpatient visits for osteoarthritis per IQR increase in ambient air pollutants at different lag days in 192 Chinese cities, 2013-2017, using ambient air pollution data from the high-resolution air quality reanalysis dataset for China.

Figure S9. Overall percent changes and 95% confidence intervals in daily outpatient visits for osteoarthritis with per IQR increase in 24h average O_3 at different lag days in 192 Chinese cities, 2013–2017

Supplementary definition of the formula

Formula (S1). City-specific associations estimate between ambient air pollutants and outpatient visits for OA and its major five subtypes at different lag days:

$$logit(P) = \alpha_{stratum\ i} + \beta pollutants_t + ns(Temp, 6) + ns(RH, 3) + Holiday$$

where *P* is the probability of outpatient visits for OA or its subtypes; α represents the vector of coefficients for stratum i; $\mathrm{pollutants_t}$ represents average air pollution concentrations at different time windows, including single lag days (the exposures of the current day and 1-2 days before outpatient visit, denoted as lag0 to lag2) and cumulative lag days (including the moving averages of exposures of the current day and previous 1-2 days to ambient air pollutants, denoted as lag01 to lag02); $\mathrm{ns(Temp, 6)}$ is the natural cubic function of the 7-day moving average temperature with 6 degree-of-freedom values (dfs); $\mathrm{ns(RH, 3)}$ is the natural cubic function of the 7-day moving average relative humidity with 3 dfs; and Holiday is a binary variable indicating whether the date was a public holiday.

Formula (S2). The attributable numbers (ANs) and attributable fractions (AFs) of outpatient visits for OA associated with short-term exposures to ambient air pollutants:

$$\mathrm{AN_i} = \frac{\mathrm{e}^{\{\log(RR)\times X_i\}} - 1}{\mathrm{e}^{\{\log(\mathrm{RR})\times \mathrm{X_i}\}}} \mathrm{N_i}$$

$$\mathrm{AF_i}\% = \frac{\Sigma_{\{i=1\}}^{m} AN \times F_i}{\mathrm{F_{\{total\}}}} \times 100\%$$

where AN_i is the AN of outpatient visits for OA in city i, and AF_i% is the AF of corresponding indicators. RR indicates the relative risk of outpatient visits per unit increment in ambient air

pollutant concentrations. X_i is the annual average concentrations of each air pollutant at the main time window in city i; N_i indicates the number of outpatient visits for OA in city i. When F_{total} represents the total number of outpatient visits, F_i is set to 1.

Formula (S3). The total excess numbers (EN) of outpatient visits for OA in association with exposure to excessive or heavily excessive ambient air pollution concentrations:

$$EN_i = (OR - 1) \times A_{admissions,i} \times H_{day,i}$$

where $A_{admissions,i}$ is the average number of daily outpatient visits for OA during the study period in city i; and $H_{day,i}$ is the number of days with excessive or heavily excessive ambient air pollution concentrations during the study period in city i.

Formula (S4). The combined effects of six major ambient air pollutants and daily outpatient visits for OA:

$$air\ pollution\ score = (\beta[PM_{2.5}] \times PM_{2.5} + \beta[PM_{10}] \times PM_{10} + \beta[NO_2] \times NO_2 + \beta[SO_2] \times SO_2 + \beta[O_3] \times O_3 + \beta[CO] \times CO) \times \left(\frac{6}{sum\ of\ the\ \beta\ coefficients}\right)$$

where the six major ambient air pollutants ars $PM_{2.5}$, PM_{10}, NO_2, SO_2, O_3, and CO; *β coefficients* is weighted by single-pollutant risk estimates.

Table S1. Comparison between ambient air pollution data from the high-resolution air quality reanalysis dataset for China and ambient air pollution data from the National Urban Air Quality Real-time Publishing Platform.

Ambient Air Pollutant	Data from the CAQRA[a]					Data from the National Urban Air Quality Real-time Publishing Platform [b]					ICC (95% CI)
	Mean	SD	IQR	Q_1	Q_{99}	Mean	SD	IQR	Q_1	Q_{99}	
$PM_{2.5}$	44.6	35.0	34.4	2.2	178.0	49.4	39.2	37.5	7.8	197.2	0.857 (0.856, 0.858)
PM_{10}	66.7	49.7	52.1	2.8	246.4	85.5	62.1	61.8	16.0	302.4	0.761 (0.759, 0.762)
NO_2	20.2	16.0	20.0	0.4	71.8	31.5	17.7	21.7	6.0	87.5	0.782 (0.781, 0.784)
SO_2	17.3	16.6	13.1	0.3	82.7	24.2	24.8	18.0	2.9	124.1	0.701 (0.699, 0.703)
8h O_3	61.1	23.6	31.8	14.7	124.9	77.7	39.3	52.2	11.0	188.6	0.713 (0.711, 0.715)
CO	0.8	0.5	0.5	0.1	2.6	1.1	4.9	0.5	0.3	3.2	0.692 (0.690, 0.694)

[a] CAQRA is the first high-resolution air quality reanalysis dataset in China that simultaneously provides the surface concentrations of six conventional air pollutants, which is of great value for many studies on the health impact assessment of air pollution and investigation of air quality changes in China, and the specific information can be obtained from https://doi.org/10.5194/essd-13-529-2021 (Kong, et al., 2020). All datasets are freely available at https://doi.org/10.11922/sciencedb.00053 (Tang, et al., 2020a).

[b] Ambient air pollution data from the National Urban Air Quality Real-time Publishing Platform before imputation.

Abbreviations: CO, carbon monoxide; ICC, intraclass correlation coefficient; IQR, interquartile range; NO_2, nitrogen dioxide; 8h O_3, 8 h maximum ozone; $PM_{2.5}$, particulate matter ≤ 2.5 μm in aerodynamic diameter; PM_{10}, particulate matter ≤ 10 μm in aerodynamic diameter; Q_1, 1st percentile; Q_{99}, 99th percentile; SD, standard deviation; SO_2, sulfur dioxide.

Table S2. Overall percent changes and 95% confidence intervals in daily outpatient visits for osteoarthritis associated with per-unit increase in ambient air pollutants at the main time window [a] in 192 Chinese cities, 2013-2017.

Air pollutant	Unit	Percent change (95% CI)
$PM_{2.5}$	10 μg/m^3	0.45 (0.32, 0.58)
PM_{10}	10 μg/m^3	0.36 (0.27, 0.45)
NO_2	10 μg/m^3	1.72 (1.47, 1.98)
SO_2	10 μg/m^3	1.85 (1.28, 2.43)
8h O_3	10 μg/m^3	0.41 (0.31, 0.52)
CO	0.1 mg/m^3	0.30 (0.17, 0.42)

[a] The main time window was lag0 for $PM_{2.5}$, PM_{10}, and 8h O_3 and lag01 for NO_2, SO_2, and CO.
Abbreviations: CI, confidence interval; CO, carbon monoxide; NO_2, nitrogen dioxide; 8h O_3, 8 h maximum ozone; $PM_{2.5}$, particulate matter ≤ 2.5 μm in aerodynamic diameter; PM_{10}, particulate matter ≤ 10 μm in aerodynamic diameter; SO_2, sulfur dioxide.

Table S3. Meta-regression results of individual and city-specific variables for the significant associations between daily outpatient visits for osteoarthritis and per-IQR increase in ambient air pollutants at the main time window [a].

	$PM_{2.5}$ (IQR: 38.26 μg/m^3)	PM_{10} (IQR: 61.00 μg/m^3)	NO_2 (IQR: 22.88 μg/m^3)	SO_2 (IQR: 18.00 μg/m^3)	8h O_3 (IQR: 49.11 μg/m^3)	CO (IQR: 0.59 mg/m^3)
	Percent change (95% CI)					
Gender						
Female	Reference					
Male	-0.23 (-0.91, 0.47)	-0.25 (-0.97, 0.48)	-0.48 (-1.36, 0.41)	-0.51 (-1.81, 0.80)	-0.22 (-1.00, 0.56)	-0.16 (-1.23, 0.92)
Age group						
<40 y	Reference					
40-64 y	-0.70 (-1.54, 0.14)	-0.86 (-1.76, 0.05)	-0.85 (-1.94, 0.26)	-0.54 (-2.18, 1.13)	1.20 (0.09, 2.32)	0.00 (-1.33, 1.34)
65-74 y	0.21 (-0.71, 1.14)	0.33 (-0.66, 1.34)	0.20 (-1.02, 1.42)	0.59 (-1.19, 2.41)	-0.51 (-1.46, 0.45)	0.64 (-0.81, 2.12)
≥75 y	0.89 (-0.10, 1.89)	1.26 (0.19, 2.35)	0.39 (-0.91, 1.70)	1.10 (-0.81, 3.03)	0.77 (-0.27, 1.83)	1.30 (-0.27, 2.90)
Season						
Cold	Reference					
Warm	-0.50 (-1.47, 0.49)	-0.37 (-1.35, 0.63)	-6.25 (-7.52, -4.97)	-2.87 (-4.74, -0.97)	-0.73 (-1.69, 0.25)	-3.13 (-4.58, -1.66)
Region						
North	Reference					
South	0.51 (-0.51, 1.53)	1.71 (0.69, 2.74)	0.79 (-0.53, 2.13)	2.06 (0.31, 3.84)	-0.49 (-1.68, 0.72)	1.40 (-0.14, 2.95)
Insurance type						
UEBMI	Reference					
URBMI	0.01 (-0.79, 0.82)	-0.56 (-1.34, 0.22)	-0.02 (-1.20, 1.17)	-0.54 (-1.91, 0.84)	0.01 (-0.79, 0.82)	-0.09 (-1.38, 1.22)
Average air pollutant concentration [b]	-0.02 (-0.06, 0.02)	-0.02 (-0.04, 0.01)	0.03 (-0.03, 0.10)	-0.04 (-0.14, 0.06)	-0.02 (-0.09, 0.04)	-0.91 (-3.45, 1.70)
Temperature (℃)	-0.07 (-0.19, 0.06)	-0.05 (-0.18, 0.08)	-0.04 (-0.17, 0.10)	-0.02 (-0.25, 0.20)	-0.03 (-0.15, 0.08)	-0.03 (-0.23, 0.17)
Relative humidity (%)	0.04 (-0.03, 0.11)	0.01 (-0.06, 0.08)	0.02 (-0.06, 0.09)	-0.01 (-0.13, 0.10)	0.02 (-0.04, 0.09)	0.02 (-0.08, 0.13)

	$PM_{2.5}$ (IQR: 38.26 μg/m^3)	PM_{10} (IQR: 61.00 μg/m^3)	NO_2 (IQR: 22.88 μg/m^3)	SO_2 (IQR: 18.00 μg/m^3)	8h O_3 (IQR: 49.11 μg/m^3)	CO (IQR: 0.59 mg/m^3)
GDP per capita (CNY 10, 000)	-0.10 (-0.24, 0.05)	-0.09 (-0.25, 0.06)	0.01 (-0.18, 0.19)	0.03 (-0.24, 0.30)	0.01 (-0.14, 0.17)	0.10 (-0.12, 0.32)

[a] The main time window was lag0 for $PM_{2.5}$, PM_{10}, and 8h O_3 and lag01 for NO_2, SO_2, and CO. *P* value <0.05 is highlighted in bold.

[b] The results for average air pollutant concentration indicate whether the association between one air pollutant and outpatient visits for osteoarthritis was modified by the average levels of the air pollutant.

Abbreviations: CI, confidence interval; CO, carbon monoxide; GDP, gross domestic product; IQR, interquartile range; NO_2, nitrogen dioxide; 8h O_3, 8 h maximum ozone; $PM_{2.5}$, particulate matter ≤ 2.5 μm in aerodynamic diameter; PM_{10}, particulate matter ≤ 10 μm in aerodynamic diameter; SO_2, sulfur dioxide; UEBMI, urban employee-based basic medical insurance scheme; URBMI, urban resident-based basic medical insurance scheme.

Table S4. Overall percent changes with 95% confidence intervals in daily outpatient visits for osteoarthritis associated with per-IQR increase in ambient air pollutants in two-pollutant models at the main time window [a] in 192 Chinese cities, 2013-2017.

	$PM_{2.5}$	PM_{10}	NO_2	SO_2	8h O_3	CO
Single-pollutant model	1.75 (1.24, 2.26)	2.26 (1.73, 2.80)	4.01 (3.41, 4.60)	3.42 (2.37, 4.49)	1.98 (1.48, 2.48)	1.87 (1.10, 2.65)
Adjusting for $PM_{2.5}$ [b]	--	--	3.75 (2.95, 4.56)	2.61 (1.48, 3.76)	1.75 (1.24, 2.27)	0.31 (-0.59, 1.21)
Adjusting for PM_{10} [b]	--	--	3.11 (2.30, 3.93)	2.12 (1.00, 3.24)	1.55 (1.03, 2.07)	-0.11 (-0.92, 0.70)
Adjusting for NO_2	0.24 (-0.38, 0.85)	0.85 (0.24, 1.47)	--	1.13 (-0.1, 2.38)	1.77 (1.26, 2.28)	-1.07 (-2.01, -0.13)
Adjusting for SO_2	0.99 (0.44, 1.55)	1.56 (0.98, 2.14)	3.69 (2.89, 4.49)	--	1.75 (1.23, 2.27)	0.60 (-0.17, 1.38)
Adjusting for 8h O_3	1.43 (0.95, 1.91)	1.94 (1.45, 2.42)	3.88 (3.27, 4.49)	2.93 (1.97, 3.89)	--	1.70 (0.98, 2.43)
Adjusting for CO	1.66 (1.03, 2.30)	2.23 (1.63, 2.83)	4.76 (3.88, 5.64)	3.18 (2.05, 4.33)	1.96 (1.44, 2.47)	--

[a] The main time window was lag0 for $PM_{2.5}$, PM_{10}, and 8h O_3 and lag01 for NO_2, SO_2, and CO.

[b] The two-pollutant model was not established for $PM_{2.5}$ and PM_{10} because of the high correlation (r=0.98) between these two particulate matter variables.

Abbreviations: CO, carbon monoxide; NO_2, nitrogen dioxide; 8h O_3, 8 h maximum ozone; $PM_{2.5}$, particulate matter ≤2.5 μm in aerodynamic diameter; PM_{10}, particulate matter ≤10 μm in aerodynamic diameter; SO_2, sulfur dioxide.

Table S5. Overall percent changes with 95% confidence intervals in daily outpatient visits for subtypes of osteoarthritis associated with per-IQR increase in ambient air pollutants in two-pollutant models at the main time window [a] in 192 Chinese cities, 2013-2017.

	$PM_{2.5}$	PM_{10}	NO_2	SO_2	8h O_3	CO
**Polyarthrosis** [b]						
Single-pollutant model	2.85 (0.97, 4.75)	3.09 (1.10, 5.11)	3.80 (2.06, 5.56)	4.18 (0.82, 7.64)	2.57 (1.02, 4.14)	1.78 (-0.31, 3.92)
Adjusting for $PM_{2.5}$	--	--	2.66 (1.04, 4.31)	2.96 (-0.24, 6.26)	2.16 (0.77, 3.58)	-2.76 (-6.78, 1.43)
Adjusting for PM_{10}	--	--	2.37 (1.05, 3.69)	2.55 (-0.31, 5.50)	2.03 (0.65, 3.42)	-2.00 (-5.57, 1.70)
Adjusting for NO_2	1.84 (-0.47, 4.21)	1.87 (-0.59, 4.38)	--	1.62 (-2.10, 5.48)	2.33 (0.68, 3.99)	-0.78 (-3.41, 1.91)
Adjusting for SO_2	2.25 (0.34, 4.21)	2.61 (0.69, 4.57)	2.76 (1.82, 3.71)	--	2.15 (0.70, 3.63)	-0.13 (-2.20, 1.97)
Adjusting for 8h O_3	2.35 (0.57, 4.17)	2.53 (0.69, 4.40)	3.55 (1.68, 5.46)	3.46 (0.43, 6.57)	--	1.32 (-0.88, 3.58)
Adjusting for CO	4.90 (1.39, 8.53)	3.85 (0.74, 7.06)	4.03 (1.95, 6.14)	4.19 (0.72, 7.79)	2.57 (1.02, 4.14)	--
Coxarthrosis [c]						
Single-pollutant model	1.48 (0.10, 2.88)	2.84 (1.64, 4.04)	4.92 (3.29, 6.57)	7.03 (3.48, 10.71)	2.45 (1.37, 3.55)	-0.68 (-3.95, 2.70)
Adjusting for $PM_{2.5}$	--	--	5.49 (3.55, 7.48)	8.40 (3.91, 13.08)	2.01 (0.89, 3.15)	-0.56 (-3.64, 2.62)
Adjusting for PM_{10}	--	--	4.23 (2.31, 6.18)	7.47 (2.91, 12.23)	2.04 (0.66, 3.44)	-2.15 (-5.61, 1.44)
Adjusting for NO_2	-1.74 (-3.06, -0.40)	-0.49 (-2.02, 1.06)	--	5.44 (1.19, 9.87)	2.34 (1.25, 3.44)	-4.49 (-8.28, -0.54)
Adjusting for SO_2	0.14 (-1.34, 1.64)	0.77 (-1.07, 2.65)	2.80 (0.11, 5.56)	--	1.94 (0.77, 3.11)	-3.31 (-6.92, 0.44)
Adjusting for 8h O_3	0.94 (-0.36, 2.26)	2.39 (1.22, 3.58)	4.21 (2.12, 6.33)	5.74 (2.24, 9.36)	--	-0.91 (-4.20, 2.49)
Adjusting for CO	1.15 (-0.28, 2.59)	2.75 (1.20, 4.33)	5.00 (3.14, 6.90)	8.2 (4.28, 12.26)	2.48 (1.38, 3.59)	--
Gonarthrosis [d]						
Single-pollutant model	2.08 (1.37, 2.79)	2.60 (1.89, 3.33)	4.32 (3.52, 5.13)	3.74 (2.32, 5.18)	2.07 (1.45, 2.69)	2.27 (1.29, 3.27)
Adjusting for $PM_{2.5}$	--	--	3.95 (2.90, 5.01)	3.09 (1.60, 4.60)	1.79 (1.15, 2.45)	0.56 (-0.61, 1.74)
Adjusting for PM_{10}	--	--	3.35 (2.28, 4.43)	2.54 (1.12, 3.99)	1.57 (0.92, 2.23)	0.12 (-0.87, 1.12)
Adjusting for NO_2	0.12 (-0.77, 1.02)	0.88 (0.04, 1.73)	--	1.59 (0.04, 3.16)	1.95 (1.34, 2.56)	-0.07 (-1.17,1.04)
Adjusting for SO_2	1.37 (0.70, 2.04)	2.00 (1.36, 2.65)	3.68 (2.85, 4.51)	--	1.76 (1.13, 2.40)	1.03 (0.04, 2.04)
Adjusting for 8h O_3	1.75 (1.06, 2.44)	2.30 (1.63, 2.98)	4.20 (3.36, 5.05)	3.38 (2.07, 4.71)	--	2.19 (1.24, 3.14)

	$PM_{2.5}$	PM_{10}	NO_2	SO_2	8h O_3	CO
Adjusting for CO	1.80 (0.81, 2.79)	2.62 (1.79, 3.46)	4.63 (3.50, 5.77)	3.39 (1.83, 4.97)	2.04 (1.42, 2.66)	--
Degeneration of cervical vertebra [e]						
Single-pollutant model	2.18 (1.27, 3.10)	2.66 (1.50, 3.83)	4.84 (3.59, 6.10)	3.44 (1.83, 5.06)	1.15 (0.23, 2.08)	2.71 (1.37, 4.06)
Adjusting for $PM_{2.5}$	--	--	4.58 (3.03, 6.16)	2.19 (0.42, 4.01)	0.70 (-0.15, 1.56)	1.17 (-0.41, 2.78)
Adjusting for PM_{10}	--	--	4.31 (2.70, 5.94)	1.73 (-0.1, 3.61)	0.57 (-0.28, 1.42)	1.01 (-0.48, 2.52)
Adjusting for NO_2	-0.16 (-0.97, 0.65)	0.19 (-1.00, 1.38)	--	0.50 (-1.22, 2.24)	0.92 (0.04, 1.81)	0.32 (-0.92, 1.58)
Adjusting for SO_2	1.55 (0.57, 2.53)	2.04 (0.75, 3.35)	5.05 (3.48, 6.65)	--	0.83 (-0.05, 1.73)	1.60 (0.22, 2.99)
Adjusting for 8h O_3	2.03 (1.21, 2.86)	2.53 (1.44, 3.63)	4.75 (3.57, 5.95)	3.17 (1.70, 4.66)	--	2.66 (1.36, 3.98)
Adjusting for CO	1.46 (0.24, 2.69)	1.98 (0.58, 3.39)	4.75 (3.11, 6.41)	2.62 (1.04, 4.23)	1.15 (0.23, 2.09)	--
Degeneration of lumbar spine [f]						
Single-pollutant model	2.83 (1.78, 3.89)	3.72 (2.51, 4.94)	4.32 (3.02, 5.64)	5.51 (3.66, 7.39)	1.54 (0.55, 2.54)	3.39 (1.72, 5.08)
Adjusting for $PM_{2.5}$	--	--	3.60 (1.95, 5.27)	4.31 (2.55, 6.11)	1.08 (0.21, 1.97)	1.49 (-0.31, 3.32)
Adjusting for PM_{10}	--	--	2.98 (1.25, 4.73)	3.60 (1.86, 5.37)	0.85 (0.02, 1.69)	0.96 (-0.77, 2.72)
Adjusting for NO_2	0.92 (-0.31, 2.18)	2.08 (0.58, 3.60)	--	3.55 (1.56, 5.57)	1.32 (0.42, 2.22)	1.00 (-0.67, 2.70)
Adjusting for SO_2	1.66 (0.60, 2.74)	2.68 (1.43, 3.94)	3.27 (1.69, 4.89)	--	1.14 (0.19, 2.10)	1.87 (0.03, 3.74)
Adjusting for 8h O_3	2.42 (1.50, 3.35)	3.35 (2.28, 4.43)	4.13 (2.89, 5.38)	5.21 (3.55, 6.91)	--	3.33 (1.70, 5.00)
Adjusting for CO	2.08 (0.81, 3.37)	3.30 (1.89, 4.73)	3.76 (2.26, 5.28)	4.68 (2.82, 6.59)	1.47 (0.49, 2.46)	--

[a] All two-pollutant models included air pollutant concentrations at the same main time window identified for corresponding subtypes.

[b] For polyarthrosiss, the main time window was lag0 for $PM_{2.5}$, PM_{10}, SO_2, 8h O_3, and CO and lag02 for NO_2.

[c] For coxarthrosis, the main time window was lag0 for $PM_{2.5}$ and PM_{10}, NO_2, SO_2, 8h O_3, and CO.

[d] For gonarthrosis, the main time window lag0 for $PM_{2.5}$, PM_{10}, and 8h O_3 and lag02 for NO_2, SO_2, and CO.

[e] For degeneration of cervical vertebra, the main time window was lag0 for $PM_{2.5}$, PM_{10}, 8h O_3, and CO and lag02 for NO_2 and SO_2.

[f] For degeneration of lumbar spine, the main time window was lag0 for $PM_{2.5}$, PM_{10}, 8h O_3, and CO and lag02 for NO_2 and SO_2.

Table S6. Sensitivity analyses for the associations between per-IQR increase in ambient air pollutant concentrations at the main time window [a] and daily outpatient visits for osteoarthritis in 192 Chinese cities, 2013–2017.

	Percent change (95% CI)			
Change in *df* of average temperature				
	df = 3	*df* = 4	*df* = 5	*df* = 6 [b]
$PM_{2.5}$	1.74 (1.23, 2.25)	1.71 (1.20, 2.22)	1.71 (1.20, 2.22)	1.73 (1.22, 2.24)
PM_{10}	2.27 (1.73, 2.80)	2.23 (1.70, 2.76)	2.2 (1.68, 2.74)	2.22 (1.69, 2.75)
NO_2	3.96 (3.37, 4.55)	3.95 (3.35, 4.54)	3.98 (3.39, 4.58)	3.98 (3.39, 4.58)
SO_2	3.42 (2.36, 4.48)	3.32 (2.28, 4.38)	3.29 (2.25, 4.34)	3.36 (2.32, 4.41)
8h O_3	2.08 (1.56, 2.61)	2.08 (1.55, 2.60)	2.04 (1.52, 2.56)	2.04 (1.52, 2.56)
CO	1.75 (0.99, 2.51)	1.69 (0.92, 2.46)	1.76 (1.01, 2.52)	1.78 (1.03, 2.53)
Change in *df* of average relative humidity				
	df = 3 [b]	*df* = 4	*df* = 5	*df* = 6
$PM_{2.5}$	1.76 (1.24, 2.27)	1.73 (1.21, 2.25)	1.74 (1.22, 2.26)	1.71 (1.20, 2.23)
PM_{10}	2.22 (1.69, 2.76)	2.22 (1.68, 2.76)	2.22 (1.68, 2.76)	2.21 (1.67, 2.74)
NO_2	4.07 (3.46, 4.69)	4.04 (3.43, 4.66)	4.04 (3.43, 4.65)	4.02 (3.42, 4.63)
SO_2	3.46 (2.43, 4.50)	3.42 (2.38, 4.46)	3.43 (2.40, 4.48)	3.43 (2.40, 4.47)
8h O_3	2.03 (1.51, 2.56)	2.06 (1.53, 2.58)	2.09 (1.56, 2.61)	2.03 (1.51, 2.55)
CO	1.91 (1.17, 2.65)	1.86 (1.11, 2.61)	1.87 (1.12, 2.63)	1.84 (1.09, 2.59)
Change in time windows of average temperature and relative humidity				
	7-day moving average [b]	14-day moving average	21-day moving average	28-day moving average
$PM_{2.5}$	1.73 (1.22, 2.24)	1.90 (1.39, 2.42)	1.89 (1.36, 2.42)	1.87 (1.33, 2.41)
PM_{10}	2.22 (1.69, 2.75)	2.40 (1.86, 2.95)	2.35 (1.81, 2.91)	2.35 (1.79, 2.91)
NO_2	3.98 (3.39, 4.58)	4.16 (3.51, 4.81)	3.90 (3.26, 4.54)	3.81 (3.17, 4.45)
SO_2	3.36 (2.32, 4.41)	3.74 (2.70, 4.78)	3.56 (2.50, 4.62)	3.65 (2.59, 4.72)
8h O_3	2.04 (1.52, 2.56)	2.23 (1.74, 2.72)	2.28 (1.78, 2.79)	2.12 (1.63, 2.61)

	Percent change (95% CI)			
CO	1.32 (0.34, 2.31)	1.75 (0.79, 2.71)	1.34 (0.43, 2.26)	1.39 (0.49, 2.29)

[a] The main time window was lag0 for $PM_{2.5}$, PM_{10}, and 8h O_3 and lag01 for NO_2, SO_2, and CO.

[b] Parameters used in the main analysis.

Abbreviations: CI, confidence interval; CO, carbon monoxide; df, degree of freedom; NO_2, nitrogen dioxide; 8h O_3, 8 h maximum ozone; $PM_{2.5}$, particulate matter ≤ 2.5 μm in aerodynamic diameter; PM_{10}, particulate matter ≤ 10 μm in aerodynamic diameter; SO_2, sulfur dioxide.

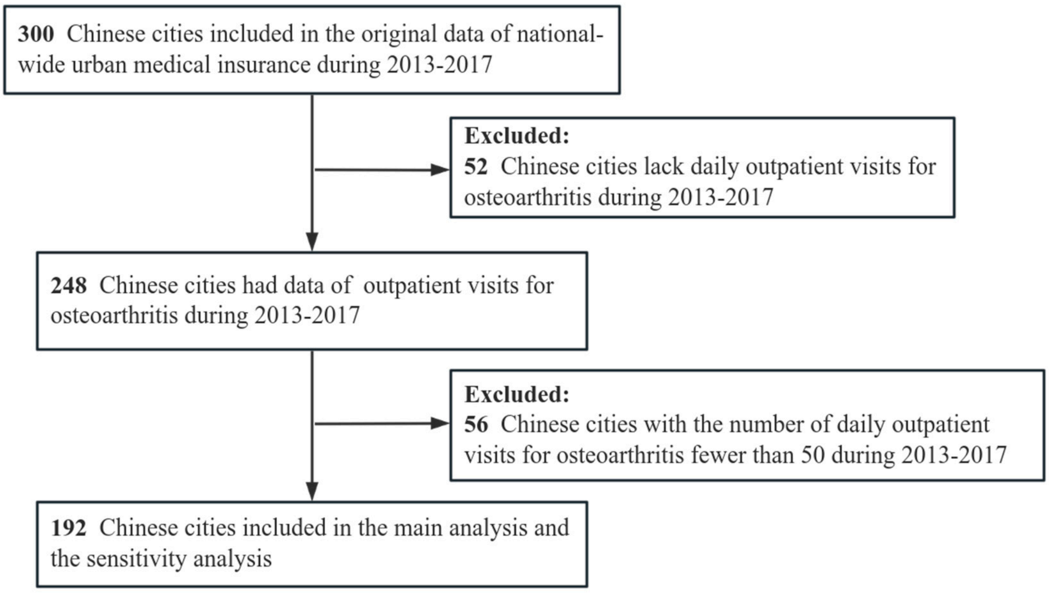

Figure S1. City selection flow chart.

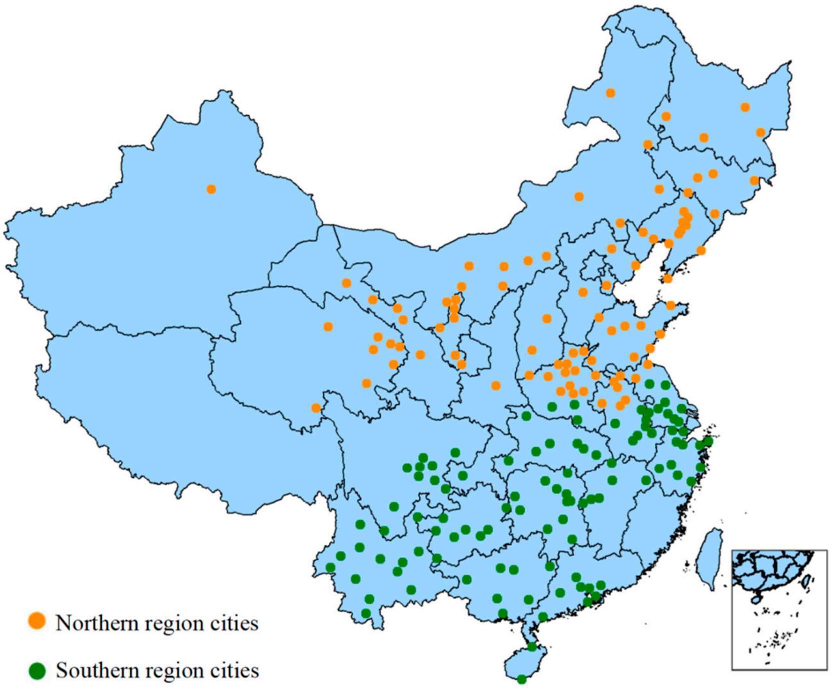

Figure S2. Central locations of the 192 Chinese cities included in the study.

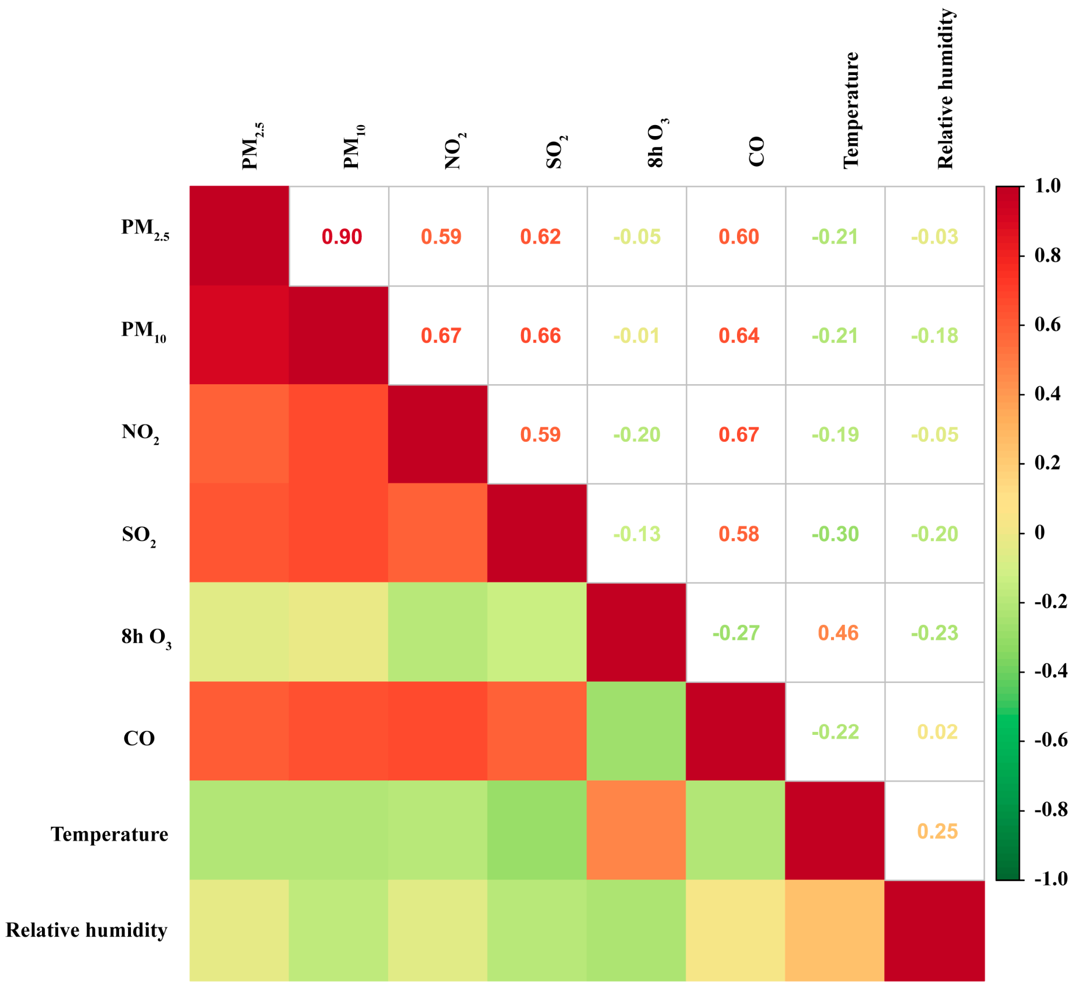

Figure S3. Spearman's correlation coefficients between ambient air pollutants and meteorological factors in 192 Chinese cities, 2013-2017.

Abbreviations: CO, carbon monoxide; NO_2, nitrogen dioxide; 8h O_3, 8 h maximum ozone; $PM_{2.5}$, particulate matter ≤ 2.5 µm in aerodynamic diameter; PM_{10}, particulate matter ≤ 10 µm in aerodynamic diameter; SO_2, sulfur dioxide.

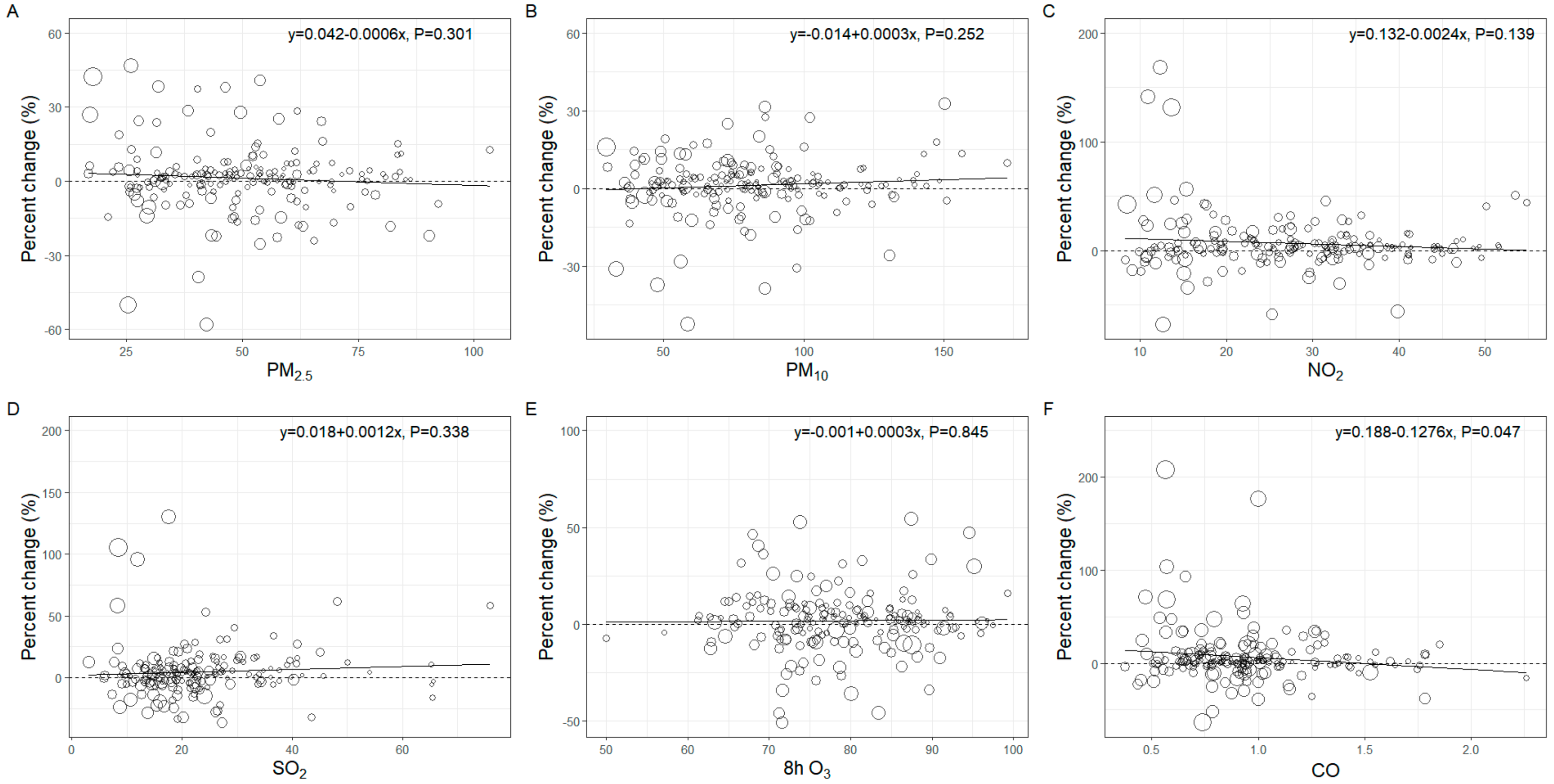

Figure S4. Scatter plots of city-specific percent changes in daily outpatient visits for osteoarthritis associated with per-IQR increase in $PM_{2.5}$ (A), PM_{10} (B), NO_2 (C), SO_2 (D), 8h O_3 (E), and CO (F) at the main time window (Y axis) versus the annual average air pollutant concentrations at the city level (X axis).

The size of circles is proportional to the standard error of the effect estimate.

Abbreviations: CO, carbon monoxide; NO_2, nitrogen dioxide; 8h O_3, 8 h maximum ozone; $PM_{2.5}$, particulate matter ≤ 2.5 µm in aerodynamic diameter; PM_{10}, particulate matter ≤ 10 µm in aerodynamic diameter; SO_2, sulfur dioxide.

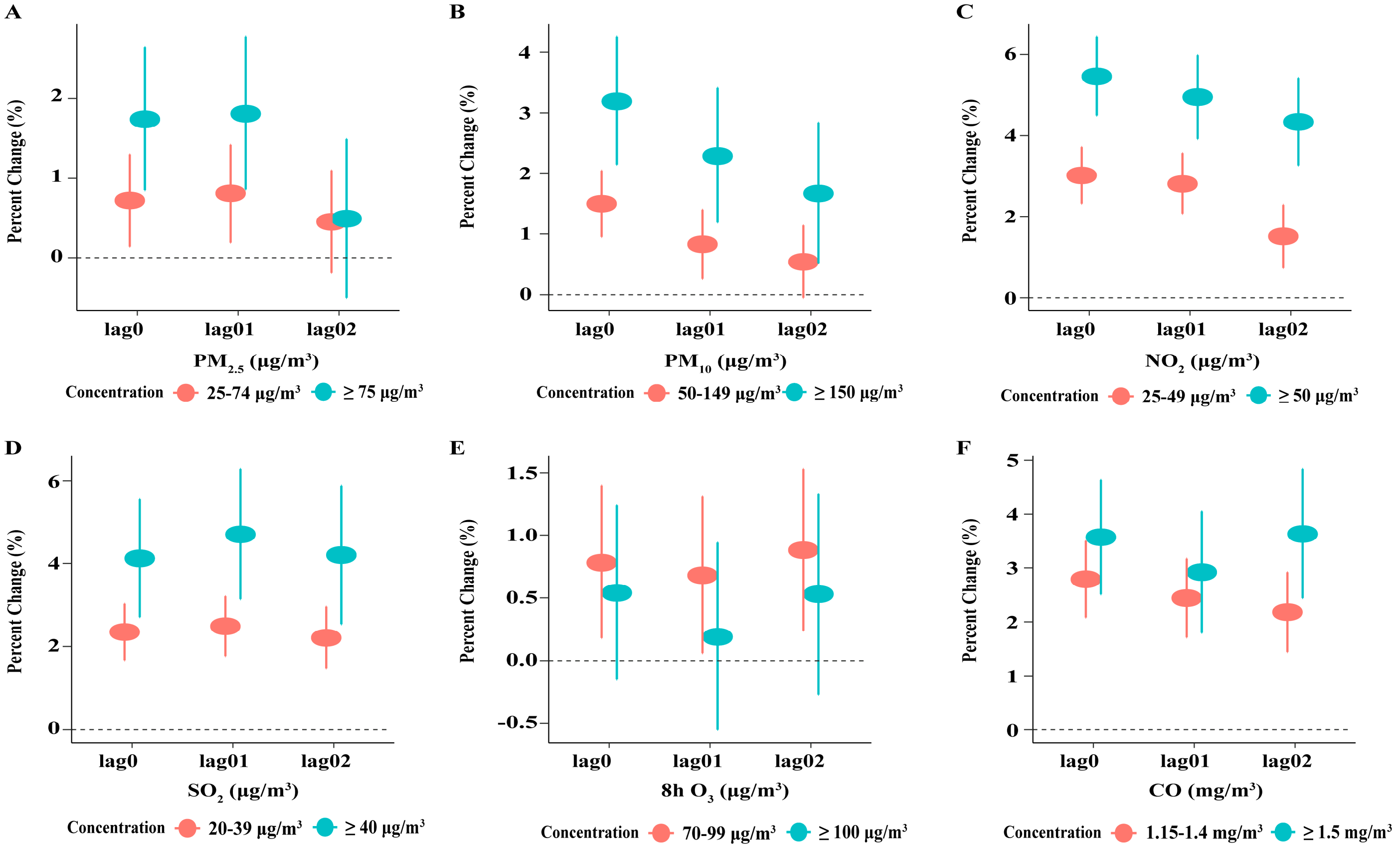

Figure S5. Overall percent changes with 95% confidence intervals in daily outpatient visits for osteoarthritis associated with excessive or heavily excessive ambient air pollutant concentrations under different definitions at different lag days in 192 Chinese cities, 2013-2017.

Abbreviations: CO, carbon monoxide; NO_2, nitrogen dioxide; 8h O_3, 8 h maximum ozone; PM_{10}, particulate matter with an aerodynamic diameter of ≤ 10 μm; $PM_{2.5}$, particulate matter with an aerodynamic diameter of ≤ 2.5 μm; SO_2, sulfur dioxide.

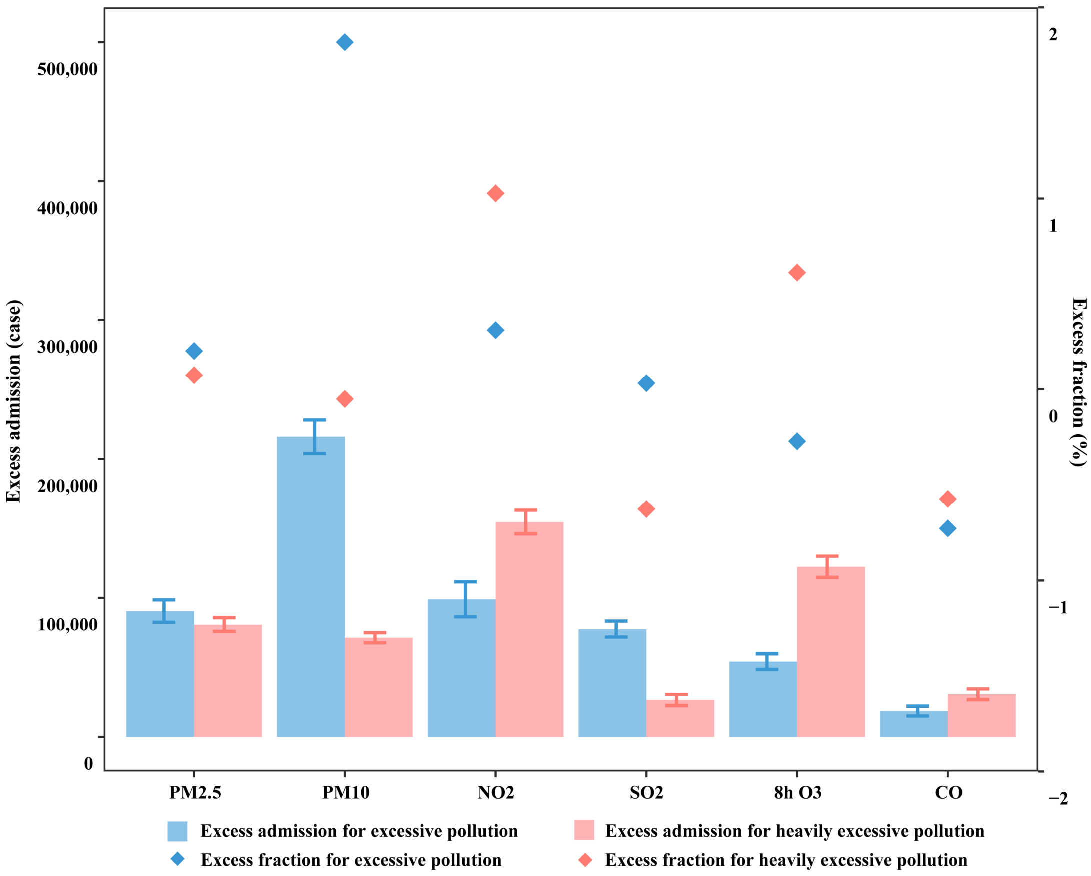

Figure S6. Excess numbers and fractions with 95% confidence intervals in outpatient visits for osteoarthritis associated with excessive or heavily excessive ambient air pollutant concentrations under different definitions [a] at the main time window [b] in 192 Chinese cities, 2013-2017.

[a] Excessive and heavily excessive $PM_{2.5}$ concentrations were defined as 25-74 μg/m^3 and ≥ 75 μg/m^3, respectively; excessive and heavily excessive PM_{10} concentrations were defined as 50-149 μg/m^3 and ≥ 150 μg/m^3, respectively; excessive and heavily excessive NO_2 concentrations were defined as 25-49 μg/m^3 and ≥ 50 μg/m^3, respectively; excessive and heavily excessive SO_2 concentrations were defined as 20-39 μg/m^3 and ≥ 40 μg/m^3, respectively; and excessive and heavily excessive CO concentrations were defined as 1.15-1.5 mg/m^3 and ≥ 1.5 mg/m^3, respectively. The reference category for each air pollutant was the days with low daily air pollution concentrations (lower than the excessive concentrations).

[b] The main time window was lag0 for $PM_{2.5}$ and PM_{10} and lag01 for NO_2, SO_2, and CO.

Abbreviations: CO, carbon monoxide; NO_2, nitrogen dioxide; PM_{10}, particulate matter with an aerodynamic diameter of ≤ 10 μm; $PM_{2.5}$, particulate matter with an aerodynamic diameter of ≤ 2.5 μm; SO_2, sulfur dioxide.

Abbreviations: Q_2, 2nd quartile; Q_3, 3rd quartile; Q_4, 4th quartile.

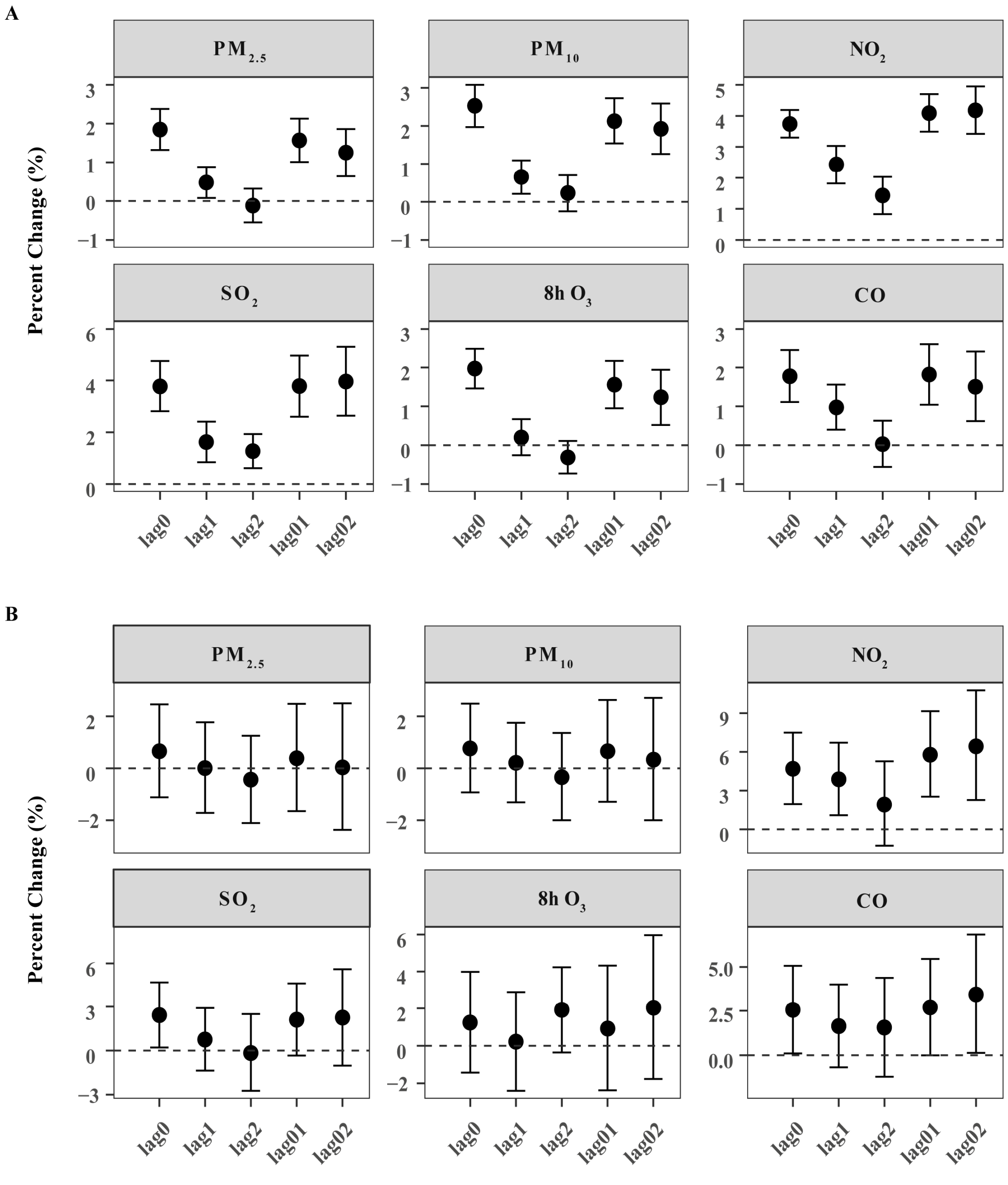

Figure S7. Overall percent changes with 95% confidence intervals in daily outpatient visits for osteoarthritis per IQR increase in ambient air pollutants at different lag days in 108 Chinese cities with 5-year data (A) and 84 Chinese cities with data for less than 5 years (B), 2013-2017. Abbreviations: CO, carbon monoxide; NO_2, nitrogen dioxide; 8h O_3, 8 h maximum ozone; $PM_{2.5}$, particulate matter ≤ 2.5 μm in aerodynamic diameter; PM_{10}, particulate matter ≤ 10 μm in aerodynamic diameter; SO_2, sulfur dioxide.

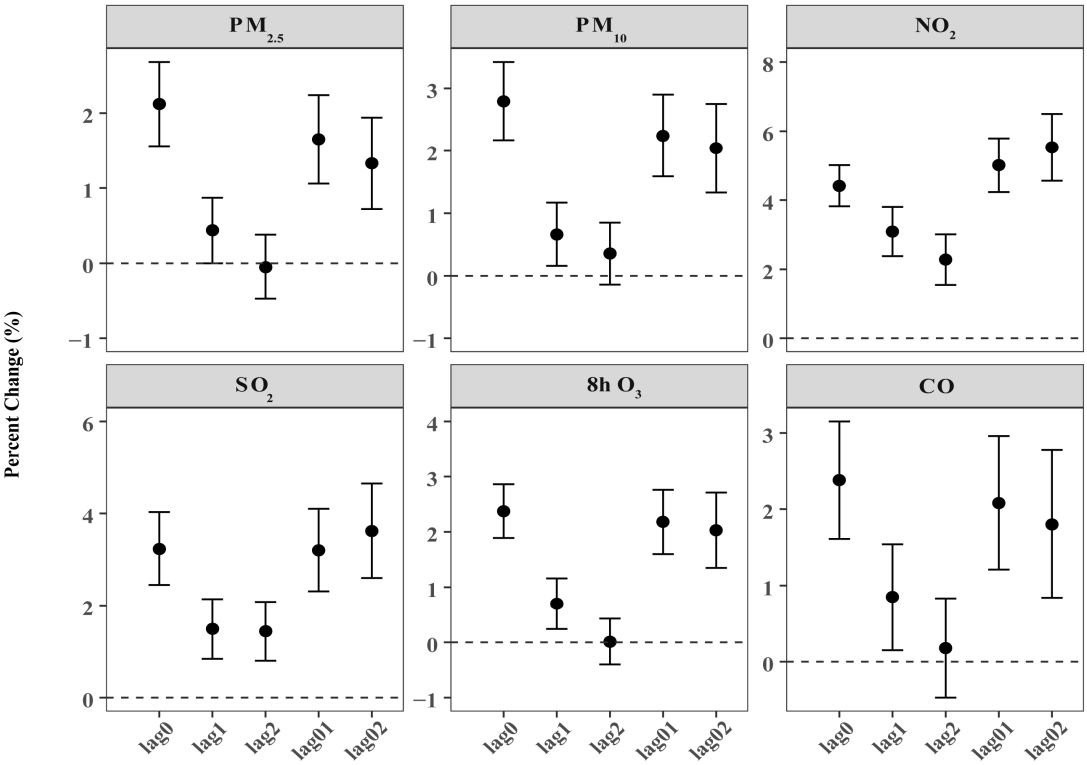

Figure S8. Overall percent changes with 95% confidence intervals in daily outpatient visits for osteoarthritis per IQR increase in ambient air pollutants at different lag days in 192 Chinese cities, 2013-2017, using ambient air pollution data from the high-resolution air quality reanalysis dataset for China.

Abbreviations: CO, carbon monoxide; NO_2, nitrogen dioxide; 8h O_3, 8 h maximum ozone; $PM_{2.5}$, particulate matter ≤ 2.5 µm in aerodynamic diameter; PM_{10}, particulate matter ≤ 10 µm in aerodynamic diameter; SO_2, sulfur dioxide.

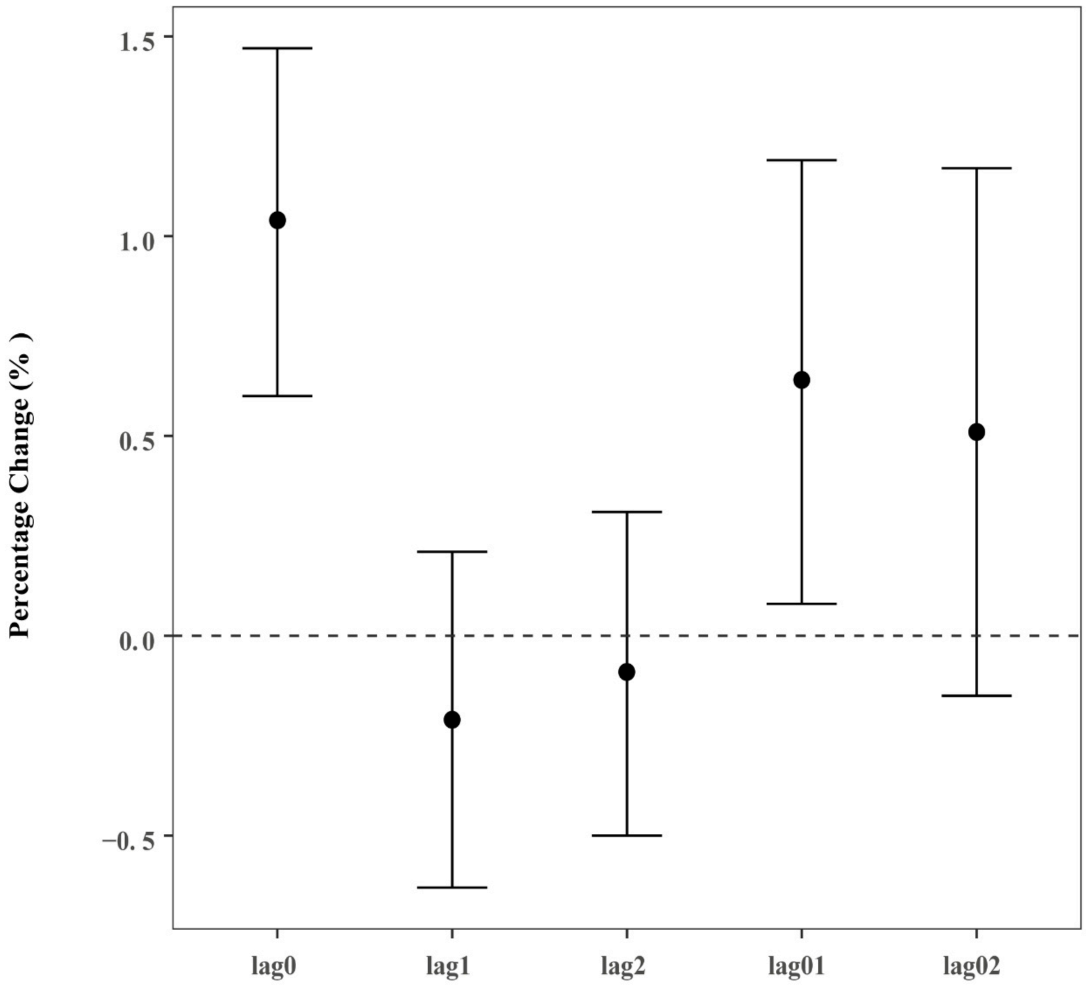

Figure S9. Overall percent changes and 95% confidence intervals in daily outpatient visits for osteoarthritis with per IQR increase in 24h average O_3 at different lag days in 192 Chinese cities, 2013–2017

Abbreviations: 24h O_3, 24 average ozone.
